# Understanding the Use of Carbon Credits by Companies: A Review of the Defining Elements of Corporate Climate Claims

**DOI:** 10.1002/gch2.202200158

**Published:** 2023-02-25

**Authors:** Danick Trouwloon, Charlotte Streck, Thiago Chagas, Glenpherd Martinus

**Affiliations:** ^1^ Climate Focus Van Diemenstraat 170 Amsterdam 1013 CP The Netherlands; ^2^ Copernicus Institute of Sustainable Development Utrecht University Heidelberglaan 2 Utrecht 3584 CS The Netherlands; ^3^ Climate Focus Schwedter Strasse 235 10119 Berlin Germany; ^4^ University of Potsdam International Relations and International Politics Am neuen Palais 10 14469 Potsdam Germany; ^5^ University of Eastern Finland UEF Law School Joensuu campus. P.O. Box 111 Kuopio FI‐80101 Finland; ^6^ Centre of Expertise Global and Inclusive Learning The Hague 2521 EN The Netherlands; ^7^ The Hague University of Applied Sciences Johanna Westerdijkplein 75 The Hague 2521 EN The Netherlands

**Keywords:** carbon credits, carbon markets, carbon offsetting, climate governance, corporate climate claims

## Abstract

Worldwide, companies are increasingly making claims about their current climate efforts and their future mitigation commitments. These claims tend to be underpinned by carbon credits issued in voluntary carbon markets to offset emissions. Corporate climate claims are largely unregulated which means that they are often (perceived to be) misleading and deceptive. As such, corporate climate claims risk undermining, rather than contributing to, global climate mitigation. This paper takes as its point of departure the proposition that a better understanding of corporate climate claims is needed to govern such claims in a manner that adequately addresses potential greenwashing risks. To that end, the paper reviews the nascent literature on corporate climate claims relying on the use of voluntary carbon credits. Drawing on the reviewed literature, three key dimensions of corporate climate claims as related to carbon credits are discussed: 1) the intended use of carbon credits: offsetting versus non‐offsetting claims; 2) the framing and meaning of headline terms: net‐zero versus carbon neutral claims; and 3) the status of the claim: future aspirational commitments versus stated achievements. The paper thereby offers a preliminary categorization of corporate climate claims and discusses risks associated with and governance implications for each of these categories.

## Graphical abstract

The nascent literature on the use of voluntary carbon credits in corporate climate claims is reviewed to offer a categorization of such claims based on three key dimensions: the intended use of carbon credits, the framing and meaning of headline terms, and the status of the claim. For each category, the paper discusses associated risks and governance implications.

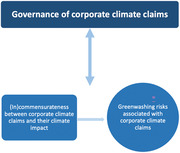

## 1 Introduction

By 1 February 2023, 8296 companies had signed up to the United Nations‐backed “Race to Zero” campaign, while the Science Based Targets initiative (SBTi) lists 4483 companies as taking climate action and 2217 companies with approved science‐based targets.^[^ ^1^, ^2^ ^]^ Corporate climate commitments are usually followed by a public announcement that the company intends to become “net‐zero”, that is, reduce and eventually eliminate its negative climate impact by a certain date, often around mid‐century.^[^ ^3^ ^]^ Many companies that are currently marketing “carbon‐neutral” products and services also claim to have already fully “neutralized” the greenhouse gas (GHG) impacts of such products.^[^ ^4^ ^]^ Most of today's corporate climate claims—not only carbon neutral and net zero, but also carbon negative, carbon free, climate neutral and climate positive—rely to a greater or lesser extent on the use of carbon credits generated from voluntary carbon markets to offset corporate emissions.^[^ ^3^, ^4^, ^5^ ^]^ This large and ever‐increasing number of claims inevitably raises the question: are such corporate climate claims accurately reflecting the efforts undertaken by companies to mitigate climate change?

Few companies release details on whether offsetting is used to complement or substitute investments into abatement of GHG emissions generated by a company's operations or within its value chain.^[^ ^3^, ^4^, ^6^, ^7^, ^8^ ^]^ The lack of transparency around climate‐related claims casts a shadow over companies’ climate strategies and their engagement in voluntary carbon markets,^[^ ^3^ ^]^ posing reputational, litigation, and regulatory risks. More worryingly, misleading and non‐authentic corporate claims can put the achievement of the temperature goals of the Paris Agreement on climate change at risk by negatively affecting capital deployment and deterring government action by making decision‐makers believe that more ambitious policies and regulation may not be needed.^[^ ^4^ ^]^

While the risks associated with corporate claims should be addressed through more robust governance, they need not necessarily discourage investments in carbon markets. On the contrary, carbon markets with high‐quality protocols, methodologies, and monitoring frameworks provide a valuable opportunity through which companies can contribute to climate change mitigation and secure the environmental integrity of emission reductions achieved. Investments in carbon markets can also have positive spill‐over (or leakage) effects by facilitating knowledge generation, technology transfer, and access to finance into regions that do not normally benefit from private investments.^[^ ^9^ ^]^ However, to fully maximize this potential, it is important that claims based on engagement in carbon markets accurately reflect the nature of that engagement.^[^ ^6^ ^]^

Despite this imperative, there is, as of yet, no consensus on what it means for a corporate claim to accurately reflect its contribution to global climate mitigation, nor is it well understood how claims can be governed to ensure that they are commensurate with global climate mitigation efforts. Indeed, while the potential of market‐based solutions to contribute to global climate change mitigation is increasingly discussed in the academic literature^[^ ^10^, ^11^, ^12^ ^]^—and methodologies for generating carbon credits are subjected to increasing academic and civil society scrutiny^[^ ^13^, ^14^, ^15^, ^16^ ^]^—voluntary carbon markets themselves and, more specifically, the claims that companies can make when they engage with such markets, continue to be insufficiently understood. Illustrating the limited public understanding of claims, a study under German consumers found that only 3% were aware of the details behind the popular “climate neutral” claim.^[^ ^17^ ^]^ This lack of both academic and public understanding of corporate climate claims hampers governance efforts to increase the transparency around such claims, leaving room for greenwashing and its associated risks for both companies and the global climate system.

While we recognize that the quality of corporate climate claims is strongly dependent on the environmental integrity of the underlying carbon credits, a discussion of the integrity of voluntary carbon credits is beyond the scope of this paper. Rather, when discussing corporate climate claims, we are explicitly assuming that such claims are underpinned by high quality voluntary carbon credits that are permanent, additional and managed to minimize leakage. Delineating our scope in this way enables us to focus our review and discussion exclusively to the understudied topic of corporate climate claims and the degree to which they accurately reflect companies’ climate mitigation efforts, thereby offering a novel contribution to the literature. For an elaborate discussion on the quality of carbon credits, we refer readers to publications that discuss the supply side integrity of carbon credit generation^[^ ^18^, ^19^ ^]^ as well as international initiatives that work on developing principles for environmentally and socially robust carbon markets, such as the Integrity Council for Voluntary Carbon Market's Core Carbon Principles.^[^ ^20^ ^]^ We further refer readers to refs. [9, 21, 22] for a discussion of the role that carbon markets can play in accelerating climate ambition.

With this paper, we seek to contribute toward a better understanding of, increased transparency around, and more robust governance of the use of carbon credits in corporate climate claims. Through a review of the nascent literature on corporate climate claims—with a particular focus on the use of carbon credits in such claims—we identify three dimensions of corporate climate claims that apply when the use of carbon credits constitutes an essential feature of the claim: 1) the intended use of carbon credits: offsetting versus non‐offsetting claims; 2) the framing and meaning of the most commonly used headline terms: net‐zero versus carbon neutral claims; and 3) the status of the claim: future aspirational commitments versus stated achievements. This enables us to make a preliminary academic contribution toward a comprehensive and user‐friendly categorization of corporate climate claims, while distilling key insights into the risks associated with and the governance implications for each of these categories, as well as identifying potential avenues for future research.

The paper is structured as follows. After this introduction, Section 2 contextualizes our analysis amidst wider discussions on the legitimacy of corporate climate claims and emerging efforts to address greenwashing risks through more robust governance. Section 3 then details our methodology by outlining the analytical framework, research questions, and search and coding strategies underpinning our review. Section 4, in turn, presents the results of our review in terms of what it means for corporate climate claims to accurately reflect climate mitigation efforts, the greenwashing risks that may emerge, as well as the associated research gaps remaining around the use of carbon credits in corporate climate claims. Section 5 discusses these results to arrive at a preliminary categorization of corporate climate claims, after which the paper is concluded in Section 6.

## 2 Contextualization

Climate‐related claims are a sub‐set of environmental claims, often made in the context of corporate social responsibility (CSR).^[^ ^23^, ^24^ ^]^ Environmental claims are assertions that companies or organizations make about environmentally beneficial attributes relevant to their operations.^[^ ^25^, ^26^ ^]^ Such claims can be made in relation to a product, a service, a brand, a process or a whole company, and may be presented as factual statements or future promises contained in sustainability reports, press releases, corporate websites, social media, labels, advertising or other marketing material.^[^ ^7^, ^27^, ^28^ ^]^

Through environmental claims, companies seek to gain legitimacy by aligning their corporate values with societal values.^[^ ^29^ ^]^ They also seek to access new markets for green products and services and seek to gain competitive advantage through green marketing strategies.^[^ ^23^, ^30^ ^]^ In this way, environmental claims serve to convince relevant stakeholders—such as consumers, shareholders, lenders, employees, policy‐makers and civil society organizations—of the reduced environmental impact of products, investments or organizations.^[^ ^24^, ^31^ ^]^

### 2.1 Carbon Offsetting and the Use of Carbon Credits in Corporate Climate Claims

Through engagement in carbon markets, companies are able to acquire carbon credits to offset (i.e., counteract or cancel out) GHG emissions for both compliance (if they have mandatory GHG reduction obligations) or voluntary purposes.^[^ ^32^, ^33^ ^]^ In such a case, a carbon credit, representing a reduction or removal of emissions of GHG, is acquired to compensate for emissions made elsewhere. A climate claim that involves offsetting requires that the “extra good” generated by offsetting is at least equivalent—in magnitude, approximate timing, and recipient population—to the original harm done.^[^ ^34^ ^]^ Only if the climate action underlying the carbon credit goes beyond the amount of GHGs emitted by the entity retiring the carbon credit, and only then, is a climate mitigation benefit provided to society in the form of lower atmospheric carbon levels.

While the voluntary use of carbon credits as offsets has enabled companies to claim carbon neutrality for brands, product lines, events, and organization for decades, carbon markets and the notion of offsetting have always been subject to principled critique. Some authors argue that the commodification and commensuration of carbon through markets is underpinned by a reductive view of nature that assumes the global carbon cycle can be measured, quantified, and parcelled up into property rights in a way that is ultimately incongruent with reality.^[^ ^35^, ^36^, ^37^, ^38^ ^]^ In addition, it is argued that through trading in GHG emissions, responsibilities that entities should perform themselves are passed onto others, a phenomenon known as the “collective sacrifice concern.”^[^ ^39^, ^40^, ^41^ ^]^ By transferring their responsibilities to third parties, original polluters need to do little to change their environmentally damaging actions, thereby alienating their civic duties.^[^ ^36^ ^]^

The role of carbon offsetting in delivering complete climate solutions is therefore widely argued to be somewhat limited.^[^ ^6^, ^39^, ^42^ ^]^ Indeed, while the act of offsetting emissions using high quality carbon credits has the same net impact on the atmosphere as the act of reducing one's own emissions an equivalent amount, meeting the temperature goals of the Paris Agreement will require that companies both reduce their emissions and offset any remaining ones at a pace much faster than currently observed.^[^ ^4^ ^]^ As such, the major risk is that offsetting offers a “cheap” license for governments, companies and individuals to continue polluting and delaying their own GHG reductions, far beyond the time frame that climate science suggests is advisable for reaching climate goals. A consensus has thus emerged that carbon offsetting should be considered an interim and supplementary measure and be carefully managed to ensure that it is complementary to—rather than replaces—other forms of public and private climate action.^[^ ^18^ ^]^ It is therefore essential to not only safeguard the supply‐side quality of carbon credits acquired—such as by assuring additionality, permanence and minimal leakage^[^ ^43^, ^44^ ^]^—but also to preserve demand‐side integrity by ensuring that the use of carbon credits complements (rather than replaces) steady emission reductions by companies within their own supply chains, thereby contributing to (rather than risking undermining) global efforts to mitigate climate change.

In this paper, we use the term commensurate to refer to alignment between the impacts of a corporate climate claim—as they manifest across all relevant dimensions, geographies and temporalities of impact—and broader, global efforts to mitigate climate change. When related to carbon credits used to address climate change, a commensurate corporate climate claim can thus be understood as one that contributes to, rather than undermines, global climate mitigation efforts.

Given our interest in climate claims made specifically by companies and organizations, our study approaches (in)commensurateness largely from a demand‐side perspective. This means that we interrogate how academic and public actors interpret such claims, as well as the role played by carbon credits vis‐à‐vis other strategies to decarbonize economies. We contend that such a focus on the interpretation of claims is important because, before corporate climate claims can contribute to global climate mitigation, they must first be interpreted by stakeholders, who must then alter their financial and governance decisions to lead to an ultimate impact. As such, even if corporate climate claims were to align with conceptual or analytical requirements, they could still diverge markedly from popular perceptions, which carries an obvious risk of unintended consequences. Similarly, it is only by considering how the use of carbon credits interacts with other decarbonization strategies to either complement or replace them, that it is possible to appraise the ultimate influence of corporate climate claims on the total package of global climate mitigation efforts. Thus, by considering the impact of corporate climate claims through the lens of (in)commensurateness, we aim to contend with, rather than ignore, the plethora of ways in which corporate climate claims have been argued to influence—be it contribute to or undermine—global climate efforts.

### 2.2 Greenwashing Risks Associated with Misleading Corporate Climate Claims

#### 2.2.1 Climate Mitigation Risks

With the increasing stakeholder preference for sustainable brands, products and services,^[^ ^30^ ^]^ some companies feel compelled to overstate their climate performance for reputational gains and increased market share^[^ ^23^ ^]^—a practice typically referred to as “greenwashing.”^[^ ^45^, ^46^, ^47^ ^]^ As definitions of greenwashing vary between stakeholders, and greenwashing is widely acknowledged to take on various forms and vary in its degree, it may be difficult to ascertain with confidence when greenwashing is taking place and whether it is indeed intentional.^[^ ^48^, ^49^ ^]^ Notwithstanding, the frontloading of climate change mitigation‐related claims which are not matched with similar ambition in tangible action may be considered “greenwashing” or “carbonwashing”, as it gives the false impression of corporate climate action being undertaken when this is in fact not the case.^[^ ^50^ ^]^ Over the last decades, greenwashing has become so common in corporate marketing practices^[^ ^51^ ^]^ that it has, according to some advertising experts, reached epidemic proportions.^[^ ^45^ ^]^ Despite the omnipresence of greenwashing, there are no studies that quantify its effect on climate action. Yet, by masking harm or delaying action, greenwashing of any degree risks seriously undermining global climate mitigation efforts in at least two ways.

First, greenwashing can extend the lifelines of “dirty businesses”^[^ ^52^ ^]^ that are inherently incompatible with the goals of the Paris Agreement. When companies misrepresent their contribution to climate change and claim to be doing more to mitigate global climate change than they truly are, this misleads consumers in their purchase decisions and investors in their financial decisions, ultimately rewarding companies who fail to take adequate action to reduce their supply chain emissions.

Second, by misrepresenting the climate impact of their practices or their efforts to address it, greenwashing by companies can deceive consumers, investors and policy makers into unwittingly accepting polluting practices,^[^ ^53^ ^]^ which leads to an overestimation of the amount of climate mitigation action taking place and an underestimation of global carbon emissions. Thinking that sufficient efforts are being made by others, stakeholders may thus feel less impetus to change their own practices. In this way, greenwashing by companies also risks undermining the climate efforts of other stakeholders.

#### 2.2.2 Business Risks

While effective oversight and scrutiny over climate‐related claims is required to ensure that corporate action contributes to—rather than undermines—global climate change mitigation, there is also a strong business case for companies to carefully craft their climate claims lest they become exposed to several business risks.

The reputational risks for companies associated with misleading claims have increased exponentially with heightened public awareness of ever more recurrent climate risks and impacts.^[^ ^23^ ^]^ The presence of civil society organizations makes it harder for companies to fake environmental engagement to increase perceived environmental legitimacy.^[^ ^29^ ^]^ A long‐term climate claim that is not credibly backed by near‐ and medium‐term targets, a robust low‐carbon transition plan, and clear explanations of how carbon credits supplement internal GHG abatement is likely to be quickly branded by civil society, academia and the media as wishful thinking or mere virtue signaling.^[^ ^47^, ^54^, ^55^, ^56^, ^57^ ^]^

The litigation and liability risks for companies have also increased in recent years,^[^ ^58^, ^59^ ^]^ as indicated by the growing number of CSR‐related lawsuits in the United States and Europe.^[^ ^60^ ^]^ These law suits are underpinned by existing consumer and investor protection rules and focus predominantly on the legal notions of “reliance” and “materiality”, that is, the extent to which corporate claims and statements were material to consumers and investors when making their purchase and investment decisions.^[^ ^58^, ^60^ ^]^ Notably, when inaccurate or unsubstantiated statements are included in official filings submitted to public authorities, the legal responsibility of companies and their overall exposure to litigation inevitably increases, as formal statements are more likely to influence investors.

From a consumer and investor protection point of view, lawsuits are beginning to emerge as a result of alleged deceptive practices that overstate environmental achievements or understate negative impacts. In August 2021, the Australasian Centre for Corporate Responsibility (ACCR) commenced legal proceedings in the Australian Federal Court against oil and gas company Santos for potentially misleading consumers and investors about the company's ability to reach net zero by 2040. ACCR argued that Santos breached Australian consumer and corporate law by lacking a clear and credible plan to carry out its announced Scope 1 and 2 long‐term targets. ACCR also affirmed that Santos had portrayed its natural gas operations as “clean energy” and thus misled the public as to the “nature, characteristics and suitability of its primary product.”^[^ ^61^ ^]^

Finally, unsubstantiated climate claims pose regulatory risks to companies, as they are increasingly triggering the scrutiny of financial, competition and consumer authorities,^[^ ^23^ ^]^ some of which have already imposed administrative sanctions. At the international level, this includes the greater use of non‐judicial grievance mechanisms for addressing unfounded and misleading corporate claims, such as OECD's grievance mechanism under the Guidelines for Multinational Enterprises. The Forest Litigation Collaborative, for instance, has lodged a complaint through the OECD Guidelines grievance mechanism against Drax, an UK power company, alleging that several of Drax's public statements related to the use of woody biomass for energy generation wrongly portray this type of biomass fuel use as “carbon neutral” and therefore mislead consumers.^[^ ^62^ ^]^ At the national level, the Dutch advertising authority has, for example, ruled that Shell's campaign offering customers the option to offset their purchases of fuel allowing them to “drive carbon neutral” is misleading.^[^ ^63^, ^64^ ^]^ Similarly, other jurisdictions are developing mandatory requirements covering both the substance and form of these broader environmental, social, and governance claims.^[^ ^65^, ^66^, ^67^ ^]^ Some countries are also preparing legislation that creates a legal duty for companies to put in place an internal due process to manage and disclose climate‐related risks.^[^ ^67^, ^68^, ^69^ ^]^ These new rules will create a new legal venue that may be used by consumers, investors, employees, and other interested stakeholders to seek redress for companies’ sustainability and climate misrepresentations.

### 2.3 Toward a Governance of Corporate Climate Claims

As the activities, inputs and processes upon which corporate climate claims are based are often internal to a company's operations and largely unobservable to outsiders, successfully addressing the risks associated with greenwashing demands robust and independent oversight over corporate climate claims. This requires full transparency over the actions backing corporate climate claims, a common set of criteria for such claims, as well as assurance and oversight over claims made by companies.

While assurance frameworks have long been in place on the supply side of the carbon market,^[^ ^114^ ^]^ for decades, the use of credits to make corporate climate claims confronted a critical governance vacuum.^[^ ^4^, ^7^ ^]^ This appears to be changing, as public, private, and hybrid models for governing corporate claims are emerging.^[^ ^70^ ^]^ For instance, authorities in several countries have issued guidance for environmental claims that expressly cover aspects related to offsetting, such as the UK Competition and Markets Authority's Green Claims Code^[^ ^71^ ^]^ and the Australian Competition and Consumer Commission's guidelines on green marketing.^[^ ^72^ ^]^ Over the course of the year 2022, both the European Union^[^ ^73^ ^]^ and the United States Securities and Exchange Commission^[^ ^74^ ^]^ have published draft reporting standards that cover disclosures that relate to the use of carbon credits. In a parallel effort, several private initiatives published standards for corporate climate claims, including the Provisional Claims Code of Practice issued by the Voluntary Carbon Market Integrity initiative (VCMI)^[^ ^75^ ^]^ and the Gold Standard Claims Guidelines.^[^ ^76^ ^]^ In turn, a hybrid governance arrangement is being convened by the International Organization for Standardization (ISO), which is constituted by national standards bodies.^[^ ^77^ ^]^ At time of writing, ISO is working on rules to standardize the use of the term “Carbon Neutrality”^[^ ^78^ ^]^ and is also collaborating with the British Standards Institute to create consensus on the definition of net‐zero targets.^[^ ^79^ ^]^

Given this rapid growth in public, private and hybrid governance arrangements around the use of carbon credits to make corporate climate claims, there is a clear imperative to improve our academic understanding of the current landscape of corporate climate claims and the associated risks, such as to serve as a basis from which claims can be transparently governed. Conversely, real‐world developments in the ever‐dynamic landscape of corporate climate claims can also helpfully inform academic research, for example by indicating where there are knowledge gaps that remain to be addressed before robust governance of corporate climate claims can be ensured. Similarly, the ways in which different countries develop regulation to address governance challenges, and the degree to which these are successful, can also inform our academic understanding of what effective climate governance looks like in different contexts. In this way, emerging climate regulation can be understood to influence the direction of academic research in much the same way that academic research seeks to inform climate governance.

## 3 Methods

### 3.1 Goals and Analytical Framework

With the aim of informing efforts to categorize and govern corporate climate claims that rely on the use of carbon credits, this paper reviews the nascent academic literature on these claims. While most upcoming governance initiatives extensively consult and collaborate with both companies and civil society organizations,^[^ ^75^, ^76^ ^]^ the speed at which the corporate climate claims landscape is developing has made it difficult for recent academic insights to be incorporated into governance efforts. Thus, by reviewing the emerging literature on corporate climate claims, we hope to offer a helpful overview of the current academic understandings of the use of carbon credits in corporate climate claims.

Our review focuses on academic articles published on this topic in the last four years (2018–2022), when voluntary carbon markets experienced a significant increase in popularity among companies. To illustrate this, the issuance of carbon credits by leading voluntary carbon market registries—including Verra's Verified Carbon Standard, the Gold Standard's SustainCert, the American Carbon Registry, the Climate Action Reserve, Plan Vivo, the Global Carbon Council and Climate Forward—increased rapidly during this period, growing almost fivefold from 75 million credits issued in 2018 to 354 million credits issued in 2021.^[^ ^80^ ^]^ This number then decreased again somewhat to 279 million in 2022.^[^ ^80^ ^]^ Thus, focusing our review on literature published between 2018 and 2022 enables us to harvest the most relevant recent academic insights on the use of carbon credits in corporate climate claims during a period of rapid growth, while the issue was largely absent in public and academic discussions before that period. Whenever relevant, insights from early, pioneering research that touch on the topic of voluntary carbon markets more generally have been incorporated to introduce and contextualize our review, as well as to discuss its findings.

### 3.2 Analytical Framework

**Figure 1:**
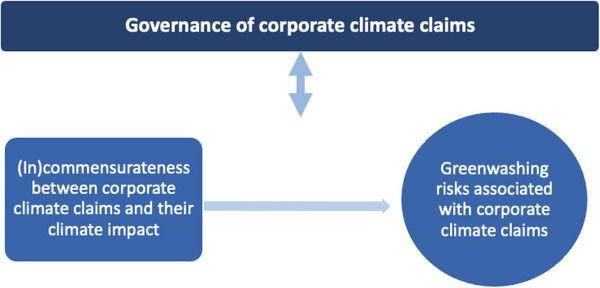
Proposed analytical framework, where the governance of corporate climate claims both aims at addressing and is informed by the greenwashing risks associated with (in)commensurate corporate climate claims.

Our review is underpinned by the contextualization offered in the previous chapter, which we operationalize into the following analytical framework. First, we consider that greenwashing occurs when corporate climate claims are not commensurate with other, global climate mitigation efforts. Second, we contend that corporate climate claims can be considered adequately and robustly governed when such claims no longer pose a greenwashing risk to business operations or to the global climate system. As such, we propose that adequate governance of corporate climate claims takes account of whether claims are commensurate with global climate mitigation efforts, such as to minimize the associated greenwashing risks. Thus, we understand the relationship between governance, on the one hand, and corporate climate claims and the associated greenwashing risks, on the other, to be a bi‐directional relationship, where appropriate governance is both informed by and able to address the risks emerging from the ever‐changing corporate climate claims landscape (**Figure** **1**).

Building on this analytical framework, our review is guided by the following research questions:

1. How are the climate claims made by companies and other organizations understood in the literature?
2. When is the use of carbon credits to achieve corporate climate goals commensurate with global efforts to mitigate climate change?
3. What are the greenwashing risks involved in using carbon credits in a way that is not commensurate with global efforts to mitigate climate change? 
4. What governance and research gaps remain around the use of carbon credits when making corporate climate claims?

### 3.3 Review Strategy

**Figure 2:**
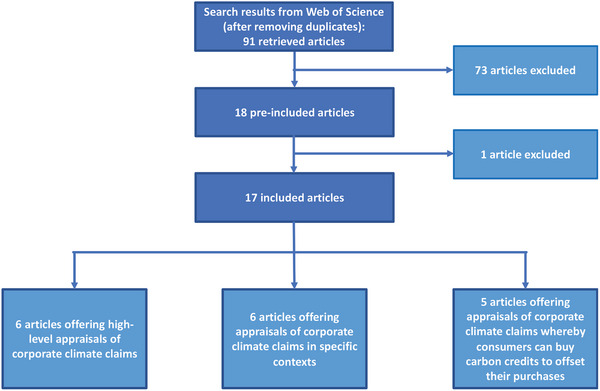
Overview of articles retrieved through the systematic component of our review.

In light of these research questions, we developed a review strategy to search for, identify, and analyze articles that discuss corporate climate claims involving the use of carbon credits. Our research interest centered on articles that provide insights into (different) understandings of commensurateness between corporate climate claims and global climate mitigation efforts, as well as the risks and governance gaps that emerge when claims are incommensurate with wider mitigation efforts. To capture relevant and recent scholarship on this rapidly evolving topic, our review strategy comprised both a systematic component and elements of an expert review. In this way, the systematic review results were complemented with insights from academic and grey literature that the authors were familiar with, and which were deemed relevant to enrich our discussion of the use of carbon credits in corporate climate claims. The following summarizes the steps followed for the systematic component of our review (**Figure** **2**). A complete overview of our methods is provided in annex 1.

Aiming at comprehensiveness, our search strategy looked for articles employing at least one term related to the carbon credits themselves (i.e., carbon credit, carbon market, or carbon offset) as well as one term explicitly relating to the use of credits in claims (i.e., net zero, carbon neutral, claim, and CSR). Net zero and carbon neutral were chosen for being the two most common terms for formulating corporate climate claims, with carbon neutrality claims being among the most popular since the inception of carbon markets and net‐zero claims rising in popularity after the Intergovernmental Panel on Climate Change (IPCC) published its Special Report on Global Warming of 1.5C in 2018.^[^ ^81^, ^82^ ^]^

We searched Web of Science for articles published between 01‐01‐2018 and 27‐03‐2022 using four search strings: 1) “Net Zero” AND (“Carbon credit*” OR “Carbon market*” OR “Carbon offset*”), 2) “Carbon Neutral” AND (“Carbon credit*” OR “Carbon market*” OR “Carbon offset*”), 3) “Claim*” AND (“Carbon credit*” OR “Carbon market*” OR “Carbon offset*”), and 4)“Corporate Social Responsibility” AND (“Carbon credit*” OR “Carbon market*” OR “Carbon offset*”) (Table S1, Supporting Information, provides an overview of the search strings and associated results). Excluding duplicates, 91 articles were retrieved through these strings.

We included in our review articles that explicitly discussed the use of carbon credits in claims made by companies and other organizations. In doing so, we excluded articles that offered a solely supply‐oriented perspective on the generation of carbon credits in the voluntary carbon market, such as articles discussing (the credibility of) carbon crediting methodologies, as well as articles discussing how carbon credit projects can be made more attractive to buyers on the voluntary carbon market, when these papers do not reflect on the resulting claims made by companies (Table S2, Supporting Information, provides an overview of the inclusion and exclusion criteria applied).

After two authors screened the titles and abstracts of all 91 articles—with frequent discussions taking place between them—18 articles appeared to match our inclusion criteria and were thus pre‐included to be read entirely. Upon complete reading, one article was found to not meet the inclusion criteria after all, leaving 17 articles for dual review. Each of these 17 articles were read in their completion and coded in parallel by two authors (Table S3, Supporting Information, provides an overview of our coding strategy).

## 4 Results

We briefly discuss here the 17 articles retrieved through our review that concern the use of carbon credits in climate claims made by companies and other organizations.

Our review yielded six articles offering high‐level appraisals of corporate carbon claims from various perspectives. This included an economic modelling exercise that analyzed why firms engage in carbon offsetting;^[^ ^23^ ^]^ a critical reflection on voluntary carbon offsetting as a mechanism for climate governance;^[^ ^83^ ^]^ and an analysis of the feasibility and credibility of carbon markets in the context of the Paris Agreement.^[^ ^4^ ^]^ In addition, the paper by Fankhauser et al.^[^ ^6^ ^]^ revisits the science behind net zero and reflects on how to make it a successful framework for corporate climate governance, while Hale et al.^[^ ^3^ ^]^ systematically appraise the robustness of the net‐zero goals of, among others, the 2000 largest publicly‐traded companies. Finally, MacCutcheon, Holmgren, and Haga^[^ ^84^ ^]^ empirically explore the relationship between consumers’ cognitive processes and their susceptibility to being misled by corporate climate claims.

Another six articles zoom in on the specific contexts wherein organizations make climate claims about their products, services, or operations. Two of these articles offer appraisals of the corporate climate claims made by a specific company: Baxter^[^ ^85^ ^]^ analyses the actions and strategies made by Sydney Airport to become carbon neutral by 2050, while Dawson, Dargusch, and Hill^[^ ^24^ ^]^ offer a detailed account of the actions undertaken and issues encountered by insurance firm Allianz in their efforts to become net zero by 2050. In a similar spirit, the paper by Helmers, Chang, and Dauwels^[^ ^86^ ^]^ assesses, compares and discusses the claimed climate impact of twenty universities worldwide. Three articles zoom in on climate claims in the building sector: Causone, Tatti, and Alongi^[^ ^87^ ^]^ advance a definition of carbon neutral buildings and identify related challenges; Shubbar et al.^[^ ^88^ ^]^ compare carbon offsetting to other carbon management strategies available for buildings; and McArthur^[^ ^89^ ^]^ adopts a multidimensional perspective to propose that carbon neutral buildings may ventilate above current code minima by offsetting GHG emissions.

The last five articles retrieved in our review deal with claims made by companies who offer consumers the option to buy carbon credits to offset their purchases, as is often done for flights. Importantly, and in contrast to the previously mentioned articles, these papers are not concerned with claims about the climate impacts of products, services or operations—as underpinned by carbon credits—but rather with an additional option offered to consumers to buy carbon credits to offset emissions associated with their purchase. Four of these articles are concerned with carbon credit claims made by airlines and either consider the ways in which such claims can be trustworthy or misleading;^[^ ^7^ ^]^ compare the likeliness that consumers opt into offsetting given different claiming strategies;^[^ ^90^, ^91^ ^]^ or explore the factors affecting consumers’ willingness to pay for carbon credits.^[^ ^92^ ^]^ In contrast, the paper by Günther et al.^[^ ^93^ ^]^ studies the willingness to pay to offset showering emissions by travellers staying in a German youth hostel, thereby also exploring how offsets may, or may not, cause travellers to take longer showers.

### 4.1 The (In)commensurateness of Corporate Climate Claims

#### 4.1.1 The Landscape of Corporate Climate Claims

The articles in our review underscore the global ubiquity of corporate climate claims. In their systematic appraisal of the 2000 largest publicly traded companies globally, Hale et al.^[^ ^3^ ^]^ find that 417 (or 21%) have made some claim about their efforts to reach net zero. These companies collectively represent nearly United States Dollars (US$)14 trillion in sales, which is 33% of the total sales of these 2000 largest companies and equivalent in size to China's GDP. Of these 417 companies, 44 (or 11%) claim to have already achieved their net zero target, with the remaining 89% of claims representing aspirational commitments.

Kreibich and Hermwille^[^ ^4^ ^]^ find a similar number (482) of companies with more than US$1 billion in revenue claiming to be pursuing or to have achieved some sort of neutrality, representing over US$16 trillion in sales combined. By turnover, the most well‐represented sectors in this global sample are finance, energy and utility, ICT, retail, and the automotive industry.^[^ ^4^ ^]^ Reflecting on the rapidly evolving nature of the climate claims landscape, the authors note that “the number of companies to be included in [their] analysis grew almost on a weekly basis.”^[^ ^4^ ^]^

To facilitate the analysis of corporate climate claims—and building on the environmental claims matrix developed by Carlson et al.^[^ ^27^ ^]^—Guix, Ollé, and Font^[^ ^7^ ^]^ propose a classification of claims according to the type of message being communicated, distinguishing between product, process, image and fact claims (**Table** **1**). When applied to the context of voluntary carbon offsets offered on airline websites, Guix, Ollé, and Font^[^ ^7^ ^]^ demonstrate that different types of claims are liable to mislead consumers in different ways. More specifically, the authors show that, when it comes to the use of voluntary carbon credits offered by airlines, image and product claims are likely to mislead by framing information that is difficult for consumers to verify in vague, broad and ambiguous ways.^[^ ^7^ ^]^ Conversely, process and fact claims were found to be more likely to be trustworthy and supported by statements that are easy to verify, such as certifications and standardizations.^[^ ^7^ ^]^

**Table 1:** Classification of corporate climate claims according to the type of message communicated

Type of environmental claim	Description of claim^a)^	Application to voluntary carbon offsets offered by airlines^b)^
Product claims	Refer to the ecological attributes of a product.	Describe the general environmental attributes of carbon offsetting or the specific characteristics of the airlines’ offsetting program.
Process claims	Refer to the ecological performance of a process or technique.	Describe aspects of the process of setting up, managing and marketing carbon offset projects, such as the methodologies used to calculate carbon offsets, third‐party certification and standard setting.
Image claims	Enhance organizations’ green image to amass public support.	Position the airline as a green firm with the aim of increasing their credibility and attracting flyers with strong climate concerns.
Fact claims	Are independent factual statements about the environment.	Offer factual information on the topic of flying or carbon offsetting.

^a)^ From Carlson et al.,^[^ ^27^ ^]^ as presented in Guix, Ollé, and Font^[^ ^7^ ^]^

^b)^ From Guix, Ollé, and Font.^[^ ^7^ ^]^

#### 4.1.2 The Use of Carbon Credits in Corporate Climate Claims

Our review illustrates the relevance of carbon credits generated on voluntary markets in corporate climate claims. Of the 482 large companies with climate pledges analyzed by Kreibich and Hermwille,^[^ ^4^ ^]^ only 36 (7%) are explicit about their intention to not use carbon credits to offset emissions, while 216 (45%) companies explicitly intend to use offsetting. Yet, despite the prominence of carbon credits in corporate climate strategies, companies remain rather opaque about this in their claims, as illustrated by the remaining 230 (48%) of the large companies analyzed by Kreibich and Hermwille^[^ ^4^ ^]^ that are ambiguous about their use of offsets. In this context, Hale et al.^[^ ^3^ ^]^ find that only 87 (21%) of the 417 large companies with climate targets in their sample have set some pre‐conditions on how to use them.

The prominence of carbon credits in claims can be attributed to the global nature of climate change—providing scope for compensation across geographies—as well as the inability of organizations to balance their own operational emissions and removals, while consumers and other stakeholders increasingly demand that they do so.^[^ ^3^, ^4^, ^6^, ^23^, ^83^ ^]^ In considering corporate carbon footprints a market externality, Bertini et al.^[^ ^23^ ^]^ further demonstrate how carbon offsetting is an economically attractive climate governance option to firms as well as governments. In this context, a distinction can be observed in whether carbon credits are used to “net out” and compensate emissions—that is, every increase in GHG emissions somewhere must be compensated through an emission reduction measured against a baseline elsewhere—or to neutralize residual emissions—that is, GHG removals are used to achieve a balance in emissions and removals of GHGs. While it was deemed sufficient to net out emissions at a transaction level in the early eras of global climate policy when voluntary carbon markets were emerging, limiting global warming to a specific temperature goal—be it 1.5 or 2C—will require emissions to net to zero at a global atmospheric rather than transactional level.^[^ ^4^, ^6^, ^83^ ^]^ Translating such a global net‐zero target into sufficiency criteria for carbon offsetting at the transaction and claims level—and determining an appropriate role of emission reductions and emission removals therein—is a subjective exercise on which consensus is yet to be achieved, as discussed in the following subsection of our results.

#### 4.1.3 The Terminology Used in Formulating Corporate Climate Claims

Several articles in our review offer insights into what it means for a company to claim that their products, services or operations are, or will by a future date be, net zero or carbon neutral. In their paper, Fankhauser et al.^[^ ^6^ ^]^ emphasize the intrinsically scientific nature of net zero as a concept, defined in relation to the global atmosphere: “if the objective is to keep the rise in global average temperatures within certain limits, physics implies that there is a finite budget of carbon dioxide that is allowed into the atmosphere, alongside other greenhouse gases. Beyond this budget, any further release must be balanced by removal into sinks.”^[^ ^6^ ^]^ Such a balance is to be achieved and maintained on multi‐decadal timescales.^[^ ^6^ ^]^

Authors also note the difficulty in operationalizing net zero as a claim. Fankhauser et al.^[^ ^6^ ^]^ observe that before net zero can be considered a useful “frame of reference” for structuring climate action, it must be translated into decarbonization pathways through “ethical judgements, social concerns, political interests, fairness dimensions, economic considerations and technology transitions.”^[^ ^6^ ^]^ In this context, net‐zero claims typically signal a state of equilibrium between the residual emissions and the emission removals of the operations of an entire company.^[^ ^4^, ^6^ ^]^ When used in this way, net zero implies that carbon credits—in this case, strictly representing emission “removals”, as emission “reductions” are ineligible—are exclusively used to neutralize those residual emissions that cannot feasibly be abated due to a lack of available technologies.^[^ ^6^ ^]^

Another frequently used term in formulating corporate climate claims is carbon neutrality, on which several authors in our review elaborate. For example, Baxter^[^ ^85^ ^]^ builds on “the established theory of carbon neutrality” to analyze the concept of carbon neutral airports. In this context, carbon neutrality claims typically refer to a certain amount of product, service, or operational GHG emissions that has been offset or compensated through carbon credits issued on voluntary carbon markets.^[^ ^85^, ^86^, ^87^, ^88^ ^]^ In contrast to net‐zero claims, the use of carbon credits for carbon neutrality claims is typically done without a clear link to a global climate mitigation target, broader company climate strategy or within supply‐chain abatement efforts—^[^ ^86^, ^87^ ^]^ although the degree to which this is the case varies (see, e.g., Baxter^[^ ^85^ ^]^).

Dawson, Dargusch, and Hill's^[^ ^24^ ^]^ empirical account of insurance firm Allianz—which claims to have achieved carbon neutrality in 2012 and has committed to becoming net zero by 2050—further elucidates this distinction in how carbon neutral and net zero are often used by companies. While Allianz claims to have achieved carbon neutrality solely through carbon offsetting, their commitment to net zero is more stringent in that, in addition to offsetting, it involves claiming to adopt a decarbonization pathway aligned with 1.5C, achieving green electricity by 2023 and phasing out coal in their investment portfolio by 2040.^[^ ^24^ ^]^

However, we find that this distinction between net‐zero and carbon‐neutrality claims is not always observed in the literature (see, e.g., Bertini et al.^[^ ^23^ ^]^ and McArthur^[^ ^89^ ^]^). For example, Hale et al.^[^ ^3^ ^]^ note that the terminology used in formulating corporate climate claims is still “in its infancy”, emphasizing how “terms are sometimes used interchangeably despite implying different climate outcomes.” As such, the authors themselves use net zero to represent “a heterogeneous array of targets.”^[^ ^3^ ^]^

### 4.2 Risks around the Use of Carbon Credits in Corporate Climate Claims

Our review corroborates that when corporate entities aim to manage their climate impact through the acquisition of carbon credits, the risks that surface are diverse and manifold. Greenwashing risks have far‐reaching consequences that threaten the social license of the voluntary carbon market as a viable impactful pathway toward global carbon emissions reduction.^[^ ^3^, ^4^, ^6^, ^83^ ^]^ By means of example, in their appraisal of the robustness of net‐zero claims, Hale et al.^[^ ^3^ ^]^ find that only 3% of companies with a net‐zero target meet all criteria for robustness. As such, several of the studies retrieved in our review point to the need to critically assess the risks faced when corporate claims fail to adequately reflect the reality of their climate impact, such as to determine whether, and if so how, voluntary carbon offsetting can be leveraged to foster climate mitigation in a manner that is advantageous for both climate and businesses.^[^ ^88^, ^89^, ^93^ ^]^

Carbon neutrality claims may be particularly likely to mislead because many consumers fail to understand the nature of offsetting and thus inaccurately estimate the impact of carbon offsetting on the total carbon footprint of their purchases.^[^ ^17^, ^84^ ^]^ Indeed, even in cases when a corporate climate claim meets the analytical requirements for a trustworthy claim, such analytical requirements could differ so fundamentally from popular interpretations of claims, that consumers may still end up being misled. Without properly taking account of the manner in which consumers and other stakeholder interpret the meaning of corporate climate claims, there is a clear risk that governance modalities may develop analytical requirements for claims that fail to meet their stated end goal.

This risk is exacerbated by the fact that climate‐related claims involving carbon offsets are often formulated with vague language.^[^ ^3^, ^7^ ^]^ In addition, the diversity of perspectives on the appropriate role of carbon credits and offsetting in global climate governance may further confuse public opinion and increase the scope for deception.^[^ ^4^, ^83^, ^89^ ^]^ The study by Guix, Ollé, and Font^[^ ^7^ ^]^ illustrates how claims can, at once, possess elements of both misleading and trustworthy communication, underscoring the complexity involved in classifying corporate claims while also emphasizing its impetus. Further complicating this is the vested interest of companies to maintain or improve their “green image” through expedient communication, on which third‐party certification of carbon credits has had no proven mitigative effect, despite consumers interpreting carbon credit certification to signal claim credibility.^[^ ^7^, ^23^, ^90^ ^]^

A concrete example of how corporate climate claims can correlate with the unintended outcome of undermining the achievement of global climate mitigation targets is the rise in consumption that can occur when consumers operate under the understanding that carbon credits have fully compensate for their consumption.^[^ ^93^ ^]^ In such cases, often referred to as “rebounding”, the framing of consumption as “green” by corporate climate claims can provide consumers with moral licensing to not just continue, but actually to increase, their harmful consumption behaviors.^[^ ^7^, ^23^, ^84^ ^]^ Rebounding thus risks making the ultimate achievement of global temperature goals more difficult and expensive, because consumers were led to believe that their direct actions were climate friendly, when in fact they were not.

A number of authors is concerned that the use of carbon credits issued on voluntary carbon markets by companies to achieve their climate targets may undermine national efforts to reach the global temperature goals of the Paris Agreement.^[^ ^4^, ^6^ ^]^ While there are no data or studies that link voluntary carbon market activities to a decrease (or increase) in climate ambition in a certain country, corporate actors may indeed claim emission reductions and removals that would have happened at a later stage through public policies. Then climate benefits are claimed twice, once toward a corporate climate target and once toward a government goal under the Paris Agreement. In such cases a corporate claim that suggests that an investment in carbon credits has lowered global emissions cannot be guaranteed, leading to an overestimation of the extent of climate action that has taken place.^[^ ^4^, ^6^, ^83^ ^]^

### 4.3 Governance Gaps around the Use of Carbon Credits in Corporate Climate Claims

Given the proliferation in corporate climate claims and their associated risks, our review points to two major governance implications for the efficacy of carbon markets as a climate mitigation pathway.

First, the reviewed literature points to a need for claims to be formulated more clearly and transparently such as to better signal the volume and nature of climate action taking place and avoid the myriad of risks associated with incommensurate corporate climate claims.^[^ ^7^, ^84^, ^92^ ^]^ Consumers that are unaware of or ignore the harmful effects of their purchasing decisions through the allure of offsetting would benefit from claims that are transparent about the role of offsetting in corporate strategies.^[^ ^3^ ^]^ For instance, claims may carry clarifying disclaimers for consumers at the point of purchase, stipulating that offsetting is an imperfect solution to global climate mitigation and that their consumption is still associated with carbon being emitted to the atmosphere.^[^ ^84^ ^]^ In addition, offering consumers real‐time feedback on their consumption levels has been found to mitigate the risk of rebounding associated with voluntary carbon offsetting.^[^ ^93^ ^]^

Second, given the breadth of consistent climate action required to reach atmospheric net zero and keep global temperatures to 1.5 or well below 2C above pre‐industrial levels, there is a need to encourage climate efforts in the short‐, medium‐, and long‐term.^[^ ^3^, ^4^, ^6^ ^]^ Rewarding only those claims that companies can credibly prove to have achieved today disincentivizes future climate mitigation efforts, while focusing only on long‐term commitments discourages front‐loaded emission reductions.^[^ ^6^ ^]^ Transparent reporting which shows incremental yet steady improvements is important to bridge the short‐ and long‐ term, while allowing companies the time required to develop their strategies, obtain agreements and implement steps.^[^ ^3^ ^]^ In addition, companies are encouraged to adopt both long‐term targets outlining their general commitments, as well as interim short‐ and medium term goals, which are to be complementary and internally consistent.^[^ ^6^ ^]^

### 4.4 Research Gaps around the Use of Carbon Credits in Corporate Climate Claims

Our review points to two research gaps around the use of carbon credits in corporate climate claims. First, there is a need to further improve the scientific understanding around many of the concepts used when formulating corporate climate claims, including through a better understanding of the socio‐political contexts in which they are manifest and a better translation into criteria that indicate when such concepts are used robustly.^[^ ^3^, ^6^ ^]^ Examples of concepts that are commonly used in relation to corporate climate claims but could benefit from further academic analysis include the very notion of offsetting itself and how this relates to the intended use of carbon credits—as well as the way such distinctions are reflected in common terminology such as net zero, carbon neutral, climate neutral, among others. Furthermore, scientific research can play an important role in assessing how companies are progressing toward their climate targets, as well as understanding and explaining the lack of transparency currently observed around the use of carbon credits in claims.^[^ ^3^ ^]^ Finally, scientific research can shed further light on how alternative climate governance arrangements may interact with carbon offsetting to influence the quality of corporate climate claims and their associated risks, including, for example, the influence of carbon pricing, the progressive increase in ambition and accounting for nationally determined contributions (NDC) under the Paris Agreement on the use of carbon markets and corporate climate claims.^[^ ^23^ ^]^

Second, given that stakeholders must interpret claims and adjust their decisions accordingly, there is a need to improve our scientific understanding of the behavioral implications of the use of carbon credits in corporate climate claims.^[^ ^7^, ^23^, ^84^, ^93^ ^]^ For example, questions remain about the (in)abilities of consumers and stakeholders to distinguish between robust and misleading claims when these involve carbon credits, the factors on which this (in)ability is contingent, and how this (in)ability ultimately affects their decision‐making processes.^[^ ^7^ ^]^ The modelling of cognitive decision‐making processes can help shed light on these questions.^[^ ^84^ ^]^ Finally, further research could point to strategies and interventions that help manage the risks that manifest when consumers are liable to misinterpret the use of carbon credits in corporate climate claims.^[^ ^93^ ^]^

## 5 Discussion: A Categorization of Corporate Climate Claims

Investments in mitigation projects driven by the desire to generate carbon credits have real‐world advantages for the countries and communities that benefit from well‐designed carbon projects and programs that would otherwise not be implemented.^[^ ^94^, ^95^, ^96^, ^97^ ^]^ Over the next decade, finance for carbon credits generation has therefore the potential to play an important role in funding sustainable development and climate mitigation projects in the Global South.^[^ ^6^ ^]^ However, our review highlighted several conceptual weaknesses and risks associated with the use of carbon credits to compensate for emissions generated at a spatial and temporal distance.^[^ ^4^, ^6^, ^83^, ^84^ ^]^

For such carbon finance to effectively contribute to global climate change mitigation, improved transparency, and more robust governance of corporate climate claims is needed. In the following, we distil three key dimensions that apply to climate‐related claims where the use of carbon credits constitutes an essential feature of the claim. We show how more clarity around each dimension contributes to the transparency around corporate climate claims, providing insights for future governance arrangements. The three key dimensions discussed here are:

1) The intended use of carbon credits: offsetting versus non‐offsetting claims
2) The framing and meaning of headline terms: net‐zero versus carbon‐neutrality claims
3) The status of the claim: aspirational commitments versus stated achievements

### 5.1 The Intended Use of Carbon Credits: Offsetting versus Non‐Offsetting Uses

To tackle the risk of double claiming associated with offsetting, an increasing number of leading organizations and market experts encourage companies to engage in voluntary carbon market transactions without using the acquired carbon credits to offset emissions that occurred within their value chains.^[^ ^98^, ^99^, ^100^, ^101^ ^]^ Instead, companies are encouraged to make use of a non‐offset (or non‐compensatory) claims to articulate and announce their contribution to global mitigation efforts. Proposed non‐compensatory claims include: “mitigation contribution”,^[^ ^102^ ^]^ “impact claim” table,^[^ ^103^ ^]^ contribution to “global carbon neutrality”,^[^ ^99^ ^]^ or “global net‐zero” through beyond value change mitigation. Carbon credits used for non‐offsetting purposes could be linked to a quantified mitigation claim, whereby companies support climate action outside of their value chains by cancelling carbon credits from standard's registries. In this case, the purchased and cancelled carbon credits are not to be counted toward the companies’ corporate net zero targets, nor should this transaction be communicated as such.^[^ ^104^, ^105^ ^]^ **Table** **2** exemplifies different types of non‐offsetting and offsetting claims.

**Table 2:** Examples of non‐offsetting and offsetting claims

Types of claims	Implications for the use of carbon credits
Non‐offsetting claims	Companies do not use the underlying GHG reduction or removal as offsets.
Contribution to a quantified GHG reduction or removal goal	Users of carbon credits purchase and retire carbon credits without using them to compensate for emissions made in their own supply chains. Instead, users may communicate that they have contributed to the achievement of a quantified mitigation or removal target, such as a national climate target set by a host country government.
Contribution to a global net zero goal	Users of carbon credits engage in the generation of carbon credits to produce an overall (net) mitigation effect. Carbon credits could be used as part of BVCM, complementing company efforts to reduce emissions within its value chain. This could entail the retirement of carbon credits akin to the required contribution to “overall mitigation in global emissions (OMGE)” under Article 6.4 (d) of the Paris Agreement.^[^ ^117^ ^]^ Such a claim would require being backed by a corresponding adjustment.
Offsetting claims	Companies use the underlying GHG reduction or removal as offsets
Offsetting claim backed by corresponding adjustments	Users of carbon credits receive a host country corresponding adjustment in the meaning of Article 6 of the Paris Agreement. Under this option, the host country will ensure that an accounting adjustment is added to its NDC covered emissions in order to exclude the mitigation benefit of that particular transaction from the host country's NDC accounting.
Offsetting claim not backed by corresponding adjustments	Users of carbon credits make a compensatory claim without a corresponding adjustment by the host country. This option reflects the fact that host countries may not be able or willing to make corresponding adjustments, as may be the case if they encourage investments into voluntary mitigation to achieve their NDCs, or if the voluntary mitigation action falls outside of the host country's NDC. Notwithstanding, offsetting claims made without a corresponding adjustment remain controversial, and susceptible to being perceived as greenwashing.^[^ ^98^, ^106^ ^]^

Generally, non‐compensatory claims avoid many of the pitfalls and risks that come with offsetting and are preferable over compensatory claims.^[^ ^4^, ^98^, ^106^ ^]^ Non‐offsetting claims circumvent the risks of double claiming and potential disincentives for corporate or government mitigation action. Such claims allow corporates to communicate “beyond value chain mitigation” (BVCM) that goes beyond the near‐ or long‐term targets of companies.^[^ ^107^ ^]^ While the non‐offsetting use of carbon credits has gained a lot of support from civil‐society^[^ ^75^, ^103^, ^105^ ^]^ and governments, it is not clear yet how much acceptance the proposed claim will find with corporate actors.

In December 2022, non‐offsetting claims also received formal recognition in the context of the negotiations of Article 6.4 of the Paris Agreement during the 27th session of the conference of the parties to the United Nations Framework Convention on Climate Change (COP‐17). Article 6.4 units that are not authorized for use against another country's NDC or other international mitigation purposes are now classified as “mitigation contribution A6.4ERs”.^[^ ^105^ ^]^ This aligns the terminology of mitigation outcomes generated under the Article 6 twith emerging proposals for the voluntary markets, which also suggest claiming ‘mitigation contributions’ (for instance^[^ ^102^, ^108^ ^]^).This links the acquisition of carbon credits to a quantified mitigation claim that further contributes to the goals of the Paris Agreement without risking the double claiming of emission reductions.

Double claiming can also be avoided through “corresponding adjustments.”^[^ ^4^, ^98^, ^106^ ^]^ Corresponding adjustments refer to a double‐entry bookkeeping procedure agreed in Paragraph 36 of decision 1/CP.21 and elaborated in the context of the rulebook for Article 6 agreed at COP‐26 in Glasgow to prevent double claiming.^[^ ^116^ ^]^ It compels the transferring country to adjust upward its NDC‐covered emissions whenever the country authorizes and transfers an Article 6 mitigation outcome for compliance purposes. These mandatory accounting adjustments represent a positive confirmation of the government of the country where a carbon project is hosted that the country will not claim the emission reductions or removals from such project against its NDC. The adjustments made by the acquiring and transferring entity therefore “correspond”, and there is no double claiming of the emissions.

Notably, the Glasgow Decision on Article 6.2 of the Paris Agreement provides guidance on the operationalization “corresponding adjustments” but does not specify whether, when and how countries should engage in Article 6, and if or how the voluntary carbon markets may be linked. Specifically, while Article 6 mandates corresponding adjustments for mitigation outcomes used to meet another NDC or to comply with other international emission reduction obligations, it does not prescribe corresponding adjustments to the voluntary carbon market.

As an increasing number of voluntary carbon market stakeholders demand corresponding adjustments when carbon credits are used to offset corporate emissions,^[^ ^4^, ^11^, ^109^ ^]^ companies are strongly encouraged to declare whether such credits are backed by a host country adjustment or not, along with its implications associated with (real or perceived) double claiming effects. This may be achieved through a clear link to the relevant electronic registry portraying the existence (or absence) of a host country authorization, followed by the actual corresponding adjustment communicated by the host country in its biennial transparency reports under the Paris Agreement. This process would ensure full transparency and readily available information to investors and regulators in relation to potential double claiming of the impacts produced by the carbon credits underpinning a particular offsetting claim.

### 5.2 The Framing and Meaning of Headline Terms: Net‐Zero versus Carbon Neutrality

Corporate climate claims contain a gamut of terminologies such as net zero, carbon neutral, carbon zero, climate positive, or carbon negative. While these concepts continue to evolve, a general (but, as discussed above, non‐definitive) distinction can be observed between net zero and carbon neutral to frame the use of carbon credits in corporate climate claims (**Table** **3**). These two concepts deserve particular attention for being the most commonly applied by corporates to date. The use of carbon or climate neutrality dates back to the early 2000s and has been the official seal of various standards such as the Carbon Neutral by Carbon Trust,^[^ ^110^ ^]^ the Carbon Neutral Protocol by the Climate Impact Partners,^[^ ^111^ ^]^ and the Climate Neutral Certified Standard.^[^ ^112^ ^]^ In turn, net zero gained particular momentum with Intergovernmental Panel on Climate Change's (IPCC) 2018 Special Report on the impacts of global warming of 1.5 °C^[^ ^81^, ^82^ ^]^ and the need to achieve net zero carbon dioxide emissions globally by 2050, followed by Science Based Targets initiative's (SBTi) updated set of target validation criteria for corporates willing to align their strategies with the latest science.^[^ ^101^, ^107^ ^]^

**Table 3:** Examples of definitions of net‐zero and carbon neutrality claims

Framing of claim	Example of definition	Implications for the use of carbon credits
Net zero	Setting corporate net‐zero targets aligned with meeting societal climate goals means 1) achieving a scale of value chain emissions reductions consistent with the depth of abatement at the point of reaching global net‐zero in 1.5 °C pathways and 2) neutralizing the impact of any residual emissions by permanently removing an equivalent volume of carbon dioxide. ‐SBTI, The Path to Net Zero (2021)	The use of carbon credits is generally aimed at ensuring no “net” emissions occur at a global level, keeping global temperatures stable at a specific level (e.g., 1.5 or 2 C above pre‐industrial levels). The use of carbon credits is limited to neutralizing emission sources that remain unabated in a specific year of a mitigation scenario that is consistent with global efforts to reach net zero. Credits must represent emission removals; emission reductions are not eligible. Carbon credits are also endorsed for interim use to achieve BVCM as part of net zero‐aligned climate action.
Carbon neutral	Companies, processes, and products become carbon neutral when they calculate their carbon emissions and compensate for what they have produced via carbon offsetting projects. Offsetting carbon emissions, in addition to avoidance and reduction, is an important step in holistic climate action. ‐Climate Partner, Carbon Neutral, what does it actually mean? (2022)	The use of carbon credits is aimed at ensuring no “net” increase in emissions occurs per transaction, which keeps global annual emissions stable while global temperatures increase. There are no constraints on the use of carbon credits beyond the general quality criteria.

Net‐zero claims typically express a commitment by a company that their entire operations will reach an equilibrium of net zero emissions by some future date, whereby carbon credits are used only to neutralize (i.e., remove from the atmosphere) residual emissions that cannot feasibly be abated due to a lack of available technologies.^[^ ^4^, ^6^ ^]^ To credibly claim to be committed to net‐zero, companies must have adopted a Paris‐aligned and science‐based target to reduce their value chain emissions at a specific rate and by a specific date and must be progressing steadily toward that goal.^[^ ^3^, ^4^, ^6^, ^7^, ^42^, ^107^ ^]^ Furthermore, companies must clearly communicate when their net‐zero claim is aspirational in nature (rather than legally binding) and when they are on a pathway to net‐zero (but are not net‐zero yet), in addition to reporting annually on their trajectory and progress.

In turn, carbon neutrality claims typically communicate that a product, service or company has a “neutral” impact on the global carbon emission levels, a feat often achieved through a substantial reliance on the use of carbon credits for offsetting purposes.^[^ ^85^, ^86^, ^87^, ^88^ ^]^ While carbon neutrality as an overarching concept refers to the global balance of emissions and removals,^[^ ^113^ ^]^ corporates tend to use a weaker definition of carbon neutrality^[^ ^113^ ^]^ that links the emissions of a company or specific products and services to emission reductions certified by voluntary carbon market programs. Given their reliance on carbon credits—and in light of their failure to incentivize a move away from business as usual and toward global net zero emissions—companies that use carbon neutral claims should inform stakeholders of i) how they define carbon neutrality and how they seek to achieve such neutrality and ii) how the carbon neutrality claim fits into the company's Paris‐aligned decarbonization pathway.

### 5.3 The Status of Claims: Aspirational Commitments versus Stated Achievements

Finally, a distinction can be made between claims that signal a commitment to future climate action (a “commitment claim”) and those that state that a particular climate impact has already been achieved (an “achievement claim”) (**Table** **4**).

Commitment claims are forward looking and ex‐ante. They are aspirational in nature and represent voluntarily set goals that a company wishes to achieve at some point in the future. Commitment claims often communicate a corporate headline target to be reached by a certain year in the medium‐ to long‐ term. However, commitments pose the risk that they will not be met despite a company's genuine efforts—a situation we may refer to as “greenwishing”—or that they are a disguise for a company taking little to no action. Commitment claims therefore carry the risk of being “cheap talk” or “symbolic management”,^[^ ^102^ ^]^ and may lead to greenwashing. Thus, to be credible, commitment claims must be, at the very least, backed by accountability mechanisms.

In contrast to commitment claims, achievement claims inform ex‐post about the successes of past behavior. Achievement claims state a particular quality already achieved and entice the market to take informed decisions accordingly. In general, these claims convey a concrete statement of fact, as opposed to a promise or aspiration to reach a goal by a future date. The most common carbon credit achievement claim is that of “carbon neutrality” made at point of sale of products, or in relation to specific brands or businesses. Due to their materiality to consumers, the risk of rebounding is particularly large for achievement claims, pointing to the importance of disclaimers.^[^ ^75^, ^84^ ^]^

Due to the volume of global climate mitigation required to keep temperatures to 1.5 or 2C above pre‐industrial levels, achievement and commitment climate claims must come in tandem and be formulated in a manner that reinforces each other. For the majority of companies and sectors, a complete balance between emissions and removals can only be realistically achieved at some point toward mid‐century. Until that net‐zero moment, a robust climate achievement claim (e.g., of carbon neutrality today) must always be accompanied by an equally robust commitment claim (e.g., with short‐, medium‐, and long‐term GHG targets to reach net zero and a detailed transition plan). At the same time, a long‐term commitment (e.g., to become net zero by 2050) can only be deemed credible and acceptable if underpinned by annual value chain GHG reductions that are in line with the announced net zero trajectory.

Notably, both commitment and achievement claims may be responsibly used by corporates that also wish to address their historical GHG emissions (for instance, since incorporation). A corporate could, for instance, communicate to its stakeholders the intention to “take responsibility” for all its historical emissions by 2040, making publicly available a detailed methodology and time‐series GHG inventory for these past emissions, along with a detailed forward‐looking roadmap of how these would be addressed in the short‐, mid‐, and long‐term 2050. Such a commitment claim would then need to be backed by regular (e.g., annual) actions and achievements that fully demonstrate the company is on track to meet this long‐term commitment through a mix of offsetting and non‐offsetting initiatives (detailing the types of activities and geographies, the amount of emissions reductions and/or removals achieved, the year in which they were achieved, the standards used to generate and audit these, and whether they are used for offsetting purposes or to contribute to a broader societal net zero goal).

**Table 4:** Description of commitment and achievement claims and their associated risks

Status of claim	Potential governance risks undermining strong claims	Description of strong claims
Commitment claims	Commitment claims so far remain largely non‐actionable, as most governments have been slow in creating the necessary legally binding framework to oversee and control them. With little downside to making future voluntary commitments that are not backed by a clear and credible implementation plan, companies will be compelled to publish such commitment claims while continuing to engage in polluting practices.	Strong commitment claims about future climate targets—such as reaching net zero by a certain date—have reasonable grounds, including concrete plans for achieving said aspirations. They are based on measured and disclosed corporate emissions and require corporates to demonstrate concrete (annual) abatement action in line with the announced decarbonization trajectory.
Achievement claims	Rewarding only those claims that companies can credibly prove to have achieved today carries the risk of disincentivizing future climate mitigation efforts. In addition, achievement claims are particularly material to consumers, and as such carry a rebounding risk.	Strong achievement claims represent real climate benefits that have been independently verified and are additional to business‐as‐usual corporate action. They are based on measured and disclosed corporate emissions and are linked to an equally strong commitment claim, concrete abatement action, and a decarbonization trajectory.

### 5.4 Understanding and Categorizing Corporate Climate Claims

**Figure 3:**
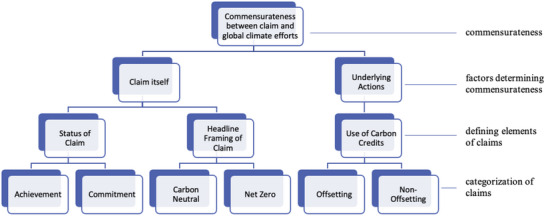
Proposed categorization of corporate climate claims and their defining elements.

Our review and ensuing discussion underscore that the commensurateness between a corporate climate claim and its climate impact depends on the quality of the policies and actions underlying said claim, as well as the framing and status of the claim itself (**Figure** **3**). Building on the three key dimensions of corporate climate claims discussed above, we propose the following categorization of claims, which can be conceived of as a preliminary taxonomy of claims aimed at fostering transparency and robust governance.^[^ ^75^, ^84^ ^]^

To be commensurate with global efforts to mitigate climate change, climate‐related claims should be underpinned by credible decarbonization pathways that are in line with climate science.^[^ ^6^, ^79^, ^101^ ^]^ How to operationalize this in practice will depend both on shared understandings of the total mitigation action necessary to reach global temperature goals, as well as on the type of corporate climate claim. Our categorization and discussion of claims allow us to observe a general distinction between achievement claims—which are typically (but, importantly, not always) carbon neutrality claims that rely substantially on offsetting—and commitment claims—which are typically (but not always) net‐zero claims that rely only to a limited extent on offsetting (**Table** **5**).^[^ ^6^, ^72^, ^92^ ^]^ The insights obtained through this categorization may inform and support emerging initiatives such as the Science‐Based Targets initiative, the VCMI, the United Nations‐backed Assessing Low Carbon Transition, and more recently the British Standards Institute‐led “our2050world”, who provide accountability frameworks for assessing companies’ headline climate targets.

**Table 5:** General distinction between commitment and achievement claims emerging from our categorization, reflecting the current landscape of corporate climate claims

Status of claim	Commitment claims	Achievement claims
Description	Aspirational and prospective statement. Expresses a future goal whose achievement is uncertain.	Factual and affirmative statement. Expresses a past goal whose accomplishment can be demonstrated.
Framing of headline claim	Most commonly framed as net zero.	Most commonly framed as carbon neutrality.
Use of carbon credits	A net‐zero commitment claim does not directly imply the use of carbon credits for compensation purposes (i.e., offsetting). If carbon credits are used for offsetting to underpin a net‐zero claim, such use is typically limited to very specific conditions and only allowed to complement robust internal abatement action.	A carbon neutrality achievement claim typically implies the use of carbon credits for compensation purposes (i.e., offsetting). When carbon credits are used for offsetting to underpin a carbon neutrality claim, there are typically no limitations on their use, beyond basic carbon credit quality criteria.
Risks	Net‐zero commitment claims are susceptible to greenwashing due to their aspirational nature. In addition, commitment claims remain largely non‐actionable due to the absence of legally binding frameworks.	Carbon neutrality achievement claims are susceptible to greenwashing as a result of rebounding, and at risk of replacing steady abatement of supply chain emissions, due to their reliance on offsetting. In addition, their reliance on offsetting puts achievement claims at risk of double claiming under the Paris Agreement.
Insights for governance	A credible net‐zero commitment claim should be transparent that a company is on a pathway to net‐zero (but has not achieved net‐zero yet) and be accompanied by transparent progress reports. A credible net‐zero commitment claim needs to be underpinned by a robust corporate strategy and transition plan containing short term targets. Investment in mitigation activities and carbon credits for non‐compensatory purposes lead to more robust claims.	A credible carbon neutrality (achievement) claim should be transparent that offsetting is an imperfect strategy for mitigating climate change, meaning no net increase in emissions rather than no net emissions at all. A credible carbon neutrality achievement claim needs to be underpinned by an equally credible net‐zero commitment claim. The risk of double claiming associated with achievement claims that relying on offsetting can be mitigated using carbon credits backed corresponding adjustment.

## 6 Conclusion

Private sector investments are pivotal for achieving the Paris Agreement temperature goal by mid‐century. In this context, voluntary carbon markets have considerable potential to bring about emission reductions and redirect climate finance to the Global South. However, the engagement of companies in voluntary carbon markets remains greatly hampered by the lack of understanding and clarity around the types of claims that can be accurately and credibly made when integrating carbon credits into corporate climate strategies.

Our review underscored the importance of transparency around the use of carbon credits, which functions as an overarching principle for all three defining elements identified in this paper. The proposed categorization of corporate climate claims has proved particularly useful in elucidating the differences between what it currently means to claim a positive climate impact that a company has already accomplished, versus signaling a future climate ambition.

Thus, we observe a general (but importantly, not absolute) distinction between those claims that signal a long‐term commitment to contributing to climate mitigation—which are typically net‐zero claims that tend to be defined over the long‐term, in line with global decarbonization pathways and without allowing offsets to substitute value‐chain emissions—and those claims that have already been achieved—which are often carbon neutrality claims, that tend to rely substantially on carbon offsetting in place of direct corporate emission reductions. As this distinction has legal and governance implications, our categorization offers a useful basis for informing the existing and emerging governance of corporate climate claims.

We are aware that our analysis offers only initial insights and preliminary steps toward a full taxonomy of standardized and transparent corporate climate‐related claims. Not taking account of the quality of carbon credits is a first limitation of our review. While most quality criteria are relevant for all corporate climate claims, additional criteria for specific sub‐categories of claims are beginning to be formulated, such as the use of carbon credits representing emission removals (rather than reduced or avoided emissions) for net‐zero claims. In addition to the research gaps that emerged from our review and are presented in Section 4, bridging the characteristics of carbon credits with the commensurateness of corporate climate claims is another important avenue for future research.

It is also important to note that our proposed demarcation between achievement‐ and commitment claims is not absolute, nor is it fixed in time. In other words, our proposal is that most (but, importantly, not all) achievement claims are currently carbon neutrality claims that rely on offsetting, whereas most commitment claims are currently net zero claims relying less on offsetting. Nevertheless, we recognize that many companies also have aspirational carbon neutrality goals, which are not yet achieved at the time they are announced, while net zero targets that are currently aspirational do allow for continuous accountability which may result in achievement over time. As the governance of claims continues to develop and corporate climate commitments are increasingly achieved, the balance between achievement‐ and commitment claims—as well as their associated features, risks, and governance gaps—is therefore likely to shift. The proposed distinction between achievement‐ and commitment claims can thus be interpreted as characteristic of the landscape of corporate climate claims at this specific moment in time when corporate climate claims are proliferating, and robust governance is only beginning to emerge. As such, we hope that this preliminary categorization of corporate climate claims and the general distinction between achievement‐ and commitment claims, as currently observed, serves as a valuable reference point for analyzing the corporate climate claims landscape as it continues to develop and becomes increasingly governed.

## Conflict of Interest

The authors declare no conflict of interest.

## Supporting information

Supporting Information
