# Supplementary material for: Understanding the Use of Carbon Credits by Companies: A Review of the Defining Elements of Corporate Climate Claims
Source: Glob Chall. 2023 Feb 25;7(4):2200158. doi: 10.1002/gch2.202200158 (PMC10069309; doi:10.1002/gch2.202200158)
Supplement: Supplementary file 1 — Supporting Information [file GCH2-7-2200158-s001.pdf]

Global Challenges

Supporting Information

for *Global Challenges,* DOI: 10.1002/gch2.202200158

Understanding the Use of Carbon Credits by Companies: A Review of the Defining Elements of Corporate Climate Claims

Danick Trouwloon, Charlotte Streck, Thiago Chagas, and Glenpherd Martinus*

Supporting Information

Supporting Information 1 – Review Methods

Danick Trouwloon, Charlotte Streck, Thiago Chagas, Glenpherd Martinus*

The analysis presented in this paper is underpinned by a review exercise aiming to synthesize insights into what it means for corporate climate claims to be (in)commensurate with global climate mitigation efforts, the risks that emerge when claims are incommensurate with their impact, as well as the associated governance and research gaps. To ensure that we capture relevant recent scholarship on this rapidly evolving topic, our review strategy comprised both a systematic component and elements of an expert review.

The search strategy guiding the systematic component of our review was designed in an iterative manner. To ensure the quality of results retrieved, the lead author explored different combinations of search terms, which were subsequently discussed among the author team. Using the final set of search strings presented in Table S1, Web of Science was searched to retrieve a total of 91 academic articles published between 01-01-2018 and 27-03-2022. A complete overview of all 91 articles is presented in Supporting Information 2.

Table S1: Overview of the number of articles retrieved during the systematic component of our review, for each combination of search terms, including duplicates.

**Search terms**	**“Net Zero”**	**“Carbon Neutral”**	**“Claim*”**	**“Corporate Social Responsibility”**
**+ “Carbon credit*”**	4	6	8	2
**+ “Carbon market*”**	8	7	11	3
**+ “Carbon offset*”**	19	26	12	7

Each of these 91 articles were screened by two authors in parallel to check whether they met our inclusion criteria. The authors met on a weekly basis to discuss the screening process and

doubts as they emerged. We included in our review English-language, peer-reviewed articles that explicitly discussed the use of carbon credits in climate claims made by companies and other organizations (Table S2). This included both articles that offered a holistic appraisal or critique of corporate carbon claims and offsetting using voluntary carbon credits, as well as articles that zoomed in on the specific contexts wherein corporate carbon claims are made, such as net-zero housing, voluntary offsetting of flight emissions by passengers, and carbon neutral universities. We excluded from our review articles that offered a solely supply-oriented perspective on the generation of carbon credits in the voluntary carbon market, such as articles discussing (the credibility of) carbon offset generation methodologies, as well as articles discussing the marketing of claims made by carbon credit projects.

Table S2: Overview of the inclusion- and exclusion criteria applied to the systematic component of our review.

Dimension	Inclusion criteria	Exclusion criteria	Decision-base
**Duplicates**	-	Always remove	Duplicates obtained from two or more search strings were identified through Zotero and removed with the “merge” command.
**Peer review**	Peer reviewed journal articles	All other types of documents, including journal articles that have not been peer reviewed, reports, conference proceedings, newspaper articles, books, book chapters, blogs, and dissertations.	The first pages of documents were screened to confirm whether they concern a journal article, followed by a Google search to confirm peer review status as necessary.
**Language**	English	All other languages, including French, Spanish, and German.	Documents were screened to confirm that the article is in English.
**Topic**	The use of carbon credits underpins	All other topics, notably: i) corporate climate	Titles, abstracts, and introductions were read

	climate claims made by companies and other organizations	claims that do not involve the use of carbon credits and ii) discussions on the voluntary carbon market and carbon credits that do not reflect on their use in corporate climate claims.	to confirm that articles are on a relevant topic.<br><br>When unsure whether article meets criteria, this was discussed with coauthors.

When unsure whether article meets criteria, this was discussed with coauthors.

After an initial screening of the 91 retrieved articles, 18 articles were preliminarily found to meet our inclusion criteria and were read in their entirety by at least two authors. During this parallel reading process, one article was found to not meet our inclusion criteria and was therefore removed from the review. The remaining 17 articles were each read and coded by two authors, using the coding strategy in Table S3.

The codes extracted from the 17 systematic review articles were used as the basis for drafting the results section and served as a primary input to our discussion and proposed classification. These results from the systematic review were complemented with insights from academic and grey literature that the authors were familiar with or which were referenced in the review articles, which we deemed to be relevant to our discussion of the use of carbon credits in corporate climate claims. In this way, elements of both a systematic review and an expert review of academic and grey literature enabled us to identify three defining elements of corporate climate claims that involve the use of carbon credits, such as to propose a preliminary categorization of these claims and offer insights for their governance.

Table S3: Overview of codes used during the full reading of included articles.

**Research question**	**Codes**	
	Written out	Shorthand
**How are climate claims made by companies and other organizations**	• Definition of claims	DEF
	• Academic understanding of claims	AC

**understood?**		
**What is the role of carbon credits in corporate climate claims?**	• Temporal scope of claims<br>• Future commitment<br>• Stated achievement	TEMP<br>TEMP_COM<br>TEMP_ACH
	• Framing of claims<br>• Net-zero<br>• Carbon neutral	FRAM<br>FRAM_NZ<br>FRAM_CN
	• Use of carbon credits in claims<br>• Offsetting<br>• Non-offsetting	USE<br>USE_OFF<br>USE_NO
**When is the use of carbon credits to achieve corporate climate goals aligned and commensurate with the claimed climate impacts?**	• Quality criteria all corporate claims	QUA_ALL
	• Quality criteria corporate climate claims	QUA_CLI
	• Quality criteria relating to the temporal scope of claims<br>• Future commitment<br>• Stated achievement	QUA_TEMP<br>QUA_TEMP_COM<br>QUA_TEMP_ACH
	• Quality criteria related to the framing of claims<br>• Net-zero<br>• Carbon neutral	QUA_FRAM<br>QUA_FRAM_NZ<br>QUA_FRAM_CN
	• Quality criteria related to the use of carbon credits in claims<br>• Offsetting<br>• Non-offsetting	QUA_USE<br>QUA_USE_OFF<br>QUA_USE_NO
**What are the risks involved in using carbon credits that are not aligned and commensurate with the claimed climate impacts?**	• Greenwashing risk	GW
	• Reputational risks	RISK_REP
	• Litigation risks	RISK_LIT
	• Regulatory risks	RISK_REG

**What governance gaps remain around the use of carbon credits for making corporate climate claims?**	• Governance of claims	GOV
**What research gaps remain around the use of carbon credits for making corporate climate claims?**	• Future research	FUTURE_RESEARCH

Supporting Information 2 – Articles retrieved during the systematic review

Danick Trouwloon, Charlotte Streck*, Thiago Chagas, Glenpherd Martinus

Aggarwal, A. 2021. "'Carbon' in Forest Carbon Projects: Evidence from India." CLIMATE AND DEVELOPMENT. https://doi.org/10.1080/17565529.2021.1956873.

Badgley, G, J Freeman, JJ Hamman, B Haya, AT Trugman, WRL Anderegg, and D Cullenward. 2022. "Systematic Over-Crediting in California's Forest Carbon Offsets Program." GLOBAL CHANGE BIOLOGY 28 (4): 1433–45. https://doi.org/10.1111/gcb.15943.

Banday, M, DR Bhardwaj, and NA Pala. 2018. "Variation of Stem Density and Vegetation Carbon Pool in Subtropical Forests of Northwestern Himalaya." JOURNAL OF SUSTAINABLE FORESTRY 37 (4): 389–402. https://doi.org/10.1080/10549811.2017.1416641.

Baxter, G. 2021. "Achieving Carbon Neutral Airport Operations By 2025: The Case of Sydney Airport, Australia." TRANSPORT AND TELECOMMUNICATION JOURNAL 22 (1): 1–14. https://doi.org/10.2478/ttj-2021-0001.

Bertini, M, S Buehler, D Halbheer, and DR Lehmann. 2022. "Carbon Footprinting and Pricing Under Climate Concerns." JOURNAL OF MARKETING 86 (2): 186–201. https://doi.org/10.1177/0022242920932930.

Bjornberg, KE. 2020. "What, If Anything, Is Wrong with Offsetting Nature?" THEORIA-A SWEDISH JOURNAL OF PHILOSOPHY 86 (6): 749–68. https://doi.org/10.1111/theo.12287.

Blackburn, J, H Mooiweer, M Parks, and A Hutson. 2018. "The Soil Value Exchange: Unlocking Nature's Value via the Market." BULLETIN OF THE ATOMIC SCIENTISTS 74 (3): 162–69. https://doi.org/10.1080/00963402.2018.1461974.

Brown, I. 2020. “Challenges in Delivering Climate Change Policy through Land Use Targets for Afforestation and Peatland Restoration.” ENVIRONMENTAL SCIENCE & POLICY 107 (May): 36–45. https://doi.org/10.1016/j.envsci.2020.02.013.

Bureau, D, A Quinet, and K Schubert. 2021. “Benefit-Cost Analysis for Climate Action.” JOURNAL OF BENEFIT-COST ANALYSIS 12 (3): 494–517. https://doi.org/10.1017/bca.2021.11.

Causone, F, A Tatti, and A Alongi. 2021. “From Nearly Zero Energy to Carbon-Neutral: Case Study of a Hospitality Building.” APPLIED SCIENCES-BASEL 11 (21). https://doi.org/10.3390/app112110148.

Cavanagh, CJ, PO Vedeld, JG Petursson, and AK Chemarum. 2021. “Agency, Inequality, and Additionality: Contested Assemblages of Agricultural Carbon Finance in Western Kenya.” JOURNAL OF PEASANT STUDIES 48 (6): 1207–27. https://doi.org/10.1080/03066150.2019.1707812.

Chai, SL, ZX Zhang, and Z Zhang. n.d. “Carbon Price Prediction for China’s ETS Pilots Using Variational Mode Decomposition and Optimized Extreme Learning Machine.” ANNALS OF OPERATIONS RESEARCH. https://doi.org/10.1007/s10479-021-04392-7.

Dawson, C, P Dargusch, and G Hill. 2022. “Assessing How Big Insurance Firms Report and Manage Carbon Emissions: A Case Study of Allianz.” SUSTAINABILITY 14 (4). https://doi.org/10.3390/su14042476.

Dong, Z, K Zhao, YQ Liu, and J Ge. 2021. “Performance Investigation of a Net-Zero Energy Building in Hot Summer and Cold Winter Zone.” JOURNAL OF BUILDING ENGINEERING 43 (November). https://doi.org/10.1016/j.jobe.2021.103192.

Dong, ZY, JJ Yang, L Yu, R Daiyan, and R Amal. 2022. “A Green Hydrogen Credit Framework for International Green Hydrogen Trading towards a Carbon Neutral Future.” INTERNATIONAL JOURNAL OF HYDROGEN ENERGY 47 (2): 728–34. https://doi.org/10.1016/j.ijhydene.2021.10.084.

Doran-Browne, N, M Wootton, C Taylor, and R Eckard. 2018. “Offsets Required to Reduce the Carbon Balance of Sheep and Beef Farms through Carbon Sequestration in Trees and Soils.” ANIMAL PRODUCTION SCIENCE 58 (9): 1648–55. https://doi.org/10.1071/AN16438.

Dray, L, and AW Schafer. n.d. “Initial Long-Term Scenarios for COVID-19’s Impact on Aviation and Implications for Climate Policy.” TRANSPORTATION RESEARCH RECORD. https://doi.org/10.1177/03611981211045067.

Ece, M. 2021. “Creating Property out of Insecurity: Territorialization and Legitimation of REDD+ in Lindi, Tanzania.” JOURNAL OF LEGAL PLURALISM AND UNOFFICIAL LAW 53 (1): 78–102. https://doi.org/10.1080/07329113.2021.1900512.

Ehara, M, H Samejima, M Yamanoshita, Y Asada, Y Shogaki, M Yano, and K Hyakumura. 2019. “REDD plus Engagement Types Preferred by Japanese Private Firms: The Challenges and Opportunities in Relation to Private Sector Participation.” FOREST POLICY AND ECONOMICS 106 (September). https://doi.org/10.1016/j.forpol.2019.06.002.

Fankhauser, S, SM Smith, M Allen, K Axelsson, T Hale, C Hepburn, JM Kendall, et al. 2022. “The Meaning of Net Zero and How to Get It Right.” NATURE CLIMATE CHANGE 12 (1): 15–21. https://doi.org/10.1038/s41558-021-01245-w.

Fox, JE, JE Campbell, and PM Acton. 2020. “Carbon Sequestration by Reforesting Legacy Grasslands on Coal Mining Sites.” ENERGIES 13 (23). https://doi.org/10.3390/en13236340.

Frewer, T. 2021. “What Exactly Do REDD plus Projects Produce? A Materialist Analysis of Carbon Offset Production from a REDD plus Project in Cambodia.” POLITICAL GEOGRAPHY 91 (November). https://doi.org/10.1016/j.polgeo.2021.102480.

Froehlich, HE, JC Afflerbach, M Frazier, and BS Halpern. 2019. “Blue Growth Potential to Mitigate Climate Change through Seaweed Offsetting.” CURRENT BIOLOGY 29 (18): 3087-+. https://doi.org/10.1016/j.cub.2019.07.041.

Ganeshamurthy, AN, V Ravindra, TR Rupa, and RM Bhatt. 2019. “Carbon Sequestration Potential of Mango Orchards in the Tropical Hot and Humid Climate of Konkan Region, India.” CURRENT SCIENCE 116 (8): 1417–23. https://doi.org/10.18520/cs/v116/i8/1417-1423.

Guix, M, C Olle, and X Font. 2022. “Trustworthy or Misleading Communication of Voluntary Carbon Offsets in the Aviation Industry.” TOURISM MANAGEMENT 88 (February). https://doi.org/10.1016/j.tourman.2021.104430.

Gunther, SA, T Staake, S Schob, and V Tiefenbeck. 2020. “The Behavioral Response to a Corporate Carbon Offset Program: A Field Experiment on Adverse Effects and Mitigation Strategies.” GLOBAL ENVIRONMENTAL CHANGE-HUMAN AND POLICY DIMENSIONS 64 (September). https://doi.org/10.1016/j.gloenvcha.2020.102123.

Hale, T, SM Smith, R Black, K Cullen, B Fay, J Lang, and S Mahmood. 2022. "Assessing the Rapidly-Emerging Landscape of Net Zero Targets." CLIMATE POLICY 22 (1): 18–29. https://doi.org/10.1080/14693062.2021.2013155.

Helmers, E, CC Chang, and J Dauwels. 2021. "Carbon Footprinting of Universities Worldwide: Part I-Objective Comparison by Standardized Metrics." ENVIRONMENTAL SCIENCES EUROPE 33 (1). https://doi.org/10.1186/s12302-021-00454-6.

Heo, MH, and MR Kim. 2018. "Asymmetric Inter-Korean Negotiations on Forest Carbon Offset Projects: Feasibility, Stakes, and Referential Variables." PACIFIC FOCUS 33 (2): 209–36. https://doi.org/10.1111/pafo.12116.

Hossaini, N, K Hewage, and R Sadiq. 2018. "Path toward Net-Zero Buildings: A Natural Capital Assessment Framework." CLEAN TECHNOLOGIES AND ENVIRONMENTAL POLICY 20 (1): 201–18. https://doi.org/10.1007/s10098-017-1469-z.

Howson, P. 2018. "Slippery Violence in the REDD plus Forests of Central Kalimantan, Indonesia." CONSERVATION & SOCIETY 16 (2): 136–46. https://doi.org/10.4103/cs.cs_16_150.

Huang, B, K Xing, D Ness, LD Liao, K Huang, PL Xie, and JL Huang. 2022. "Rethinking Carbon-Neutral Built Environment: Urban Dynamics and Scenario Analysis." ENERGY AND BUILDINGS 255 (January). https://doi.org/10.1016/j.enbuild.2021.111672.

Huang, B, K Xing, S Pullen, and LD Liao. 2020. "Exploring Carbon Neutral Potential in Urban Densification: A Precinct Perspective and Scenario Analysis." SUSTAINABILITY 12 (12). https://doi.org/10.3390/su12124814.

Ilic, D, and JC Mollet. 2022. "Voluntary Corporate Climate Initiatives and Regulatory Threat." INTERNATIONAL ECONOMICS AND ECONOMIC POLICY 19 (1): 157–84. https://doi.org/10.1007/s10368-021-00519-0.

Irshad, K, K Habib, S Algarni, BB Saha, and B Jamil. 2019. "Sizing and Life-Cycle Assessment of Building Integrated Thermoelectric Air Cooling and Photovoltaic Wall System." APPLIED THERMAL ENGINEERING 154 (May): 302–14. https://doi.org/10.1016/j.applthermaleng.2019.03.027.

Keenor, SG, AF Rodrigues, L Mao, AE Latawiec, AR Harwood, and BJ Reid. 2021. "Capturing a Soil Carbon Economy." ROYAL SOCIETY OPEN SCIENCE 8 (4). https://doi.org/10.1098/rsos.202305.

Kingwell, R. 2021. “Agriculture’s Carbon Neutral Challenge: The Case of Western Australia.” AUSTRALIAN JOURNAL OF AGRICULTURAL AND RESOURCE ECONOMICS 65 (3): 566–95. https://doi.org/10.1111/1467-8489.12440.

Kitamura, Y, S Karkour, Y Ichisugi, and N Itsubo. 2020. “Carbon Footprint Evaluation of the Business Event Sector in Japan.” SUSTAINABILITY 12 (12). https://doi.org/10.3390/su12125001.

Kohl, M, HP Ehrhart, M Knauf, and PR Neupane. 2020. “A Viable Indicator Approach for Assessing Sustainable Forest Management in Terms of Carbon Emissions and Removals.” ECOLOGICAL INDICATORS 111 (April). https://doi.org/10.1016/j.ecolind.2019.106057.

Kreibich, N, and L Hermwille. 2021. “Caught in between: Credibility and Feasibility of the Voluntary Carbon Market Post-2020.” CLIMATE POLICY 21 (7): 939–57. https://doi.org/10.1080/14693062.2021.1948384.

Kuiti, MR, D Ghosh, P Basu, and A Bisi. 2020. “Do Cap-and-Trade Policies Drive Environmental and Social Goals in Supply Chains: Strategic Decisions, Collaboration, and Contract Choices.” INTERNATIONAL JOURNAL OF PRODUCTION ECONOMICS 223 (May). https://doi.org/10.1016/j.ijpe.2019.107537.

Lee, DH, DH Kim, and SI Kim. 2018. “Characteristics of Forest Carbon Credit Transactions in the Voluntary Carbon Market.” CLIMATE POLICY 18 (2): 235–45. https://doi.org/10.1080/14693062.2016.1277682.

Lehmann, I. 2019. “When Cultural Political Economy Meets ‘charismatic Carbon’ Marketing: A Gender-Sensitive View on the Limitations of Gold Standard Cookstove Offset Projects.” ENERGY RESEARCH & SOCIAL SCIENCE 55 (September): 146–54. https://doi.org/10.1016/j.erss.2019.05.001.

Lima, GC de, ALL Toledo, and L Bourikas. 2021. “The Role of National Energy Policies and Life Cycle Emissions of PV Systems in Reducing Global Net Emissions of Greenhouse Gases.” ENERGIES 14 (4). https://doi.org/10.3390/en14040961.

Lin, R, JX Guo, XJ Li, P Patel, and A Seifitokaldani. 2020. “Electrochemical Reactors for CO2 Conversion.” CATALYSTS 10 (5). https://doi.org/10.3390/catal10050473.

Liu, HY. 2019. “Building a Dwelling That Remains Carbon-Neutral over Its Lifetime - A Case Study in Kinmen.” JOURNAL OF CLEANER PRODUCTION 208 (January): 522–29. https://doi.org/10.1016/j.jclepro.2018.10.101.

Liu, Y, QQ Jiang, and R Gleasure. 2021. "Hitting Net-Zero Without Stopping Flying: Increasing Air Travelers' Likelihood to Opt-in to Voluntary Carbon Offsetting." JOURNAL OF TRAVEL RESEARCH. https://doi.org/10.1177/00472875211057607.

Ma, R, and HQ Qian. n.d. "Plant-Level Evaluation Of China's National Emissions Trading Scheme: Benchmark Matters." CLIMATE CHANGE ECONOMICS. https://doi.org/10.1142/S2010007822400097.

MacCutcheon, D, M Holmgren, and A Haga. 2020. "Assuming the Best: Individual Differences in Compensatory 'Green' Beliefs Predict Susceptibility to the Negative Footprint Illusion." SUSTAINABILITY 12 (8). https://doi.org/10.3390/su12083414.

Machado, PG, A Hawkes, and CD Ribeiro. 2021. "What Is the Future Potential of CCS in Brazil? An Expert Elicitation Study on the Role of CCS in the Country." INTERNATIONAL JOURNAL OF GREENHOUSE GAS CONTROL 112 (December). https://doi.org/10.1016/j.ijggc.2021.103503.

Maraseni, T, and K Reardon-Smith. 2019. "Meeting National Emissions Reduction Obligations: A Case Study of Australia." ENERGIES 12 (3). https://doi.org/10.3390/en12030438.

Marino, BDV, and N Bautista. 2022. "Commercial Forest Carbon Protocol Over-Credit Bias Delimited by Zero-Threshold Carbon Accounting." TREES FORESTS AND PEOPLE 7 (March). https://doi.org/10.1016/j.tfp.2021.100171.

Marino, BDV, M Mincheva, and A Doucett. 2019. "California Air Resources Board Forest Carbon Protocol Invalidates Offsets." PEERJ 7 (September). https://doi.org/10.7717/peerj.7606.

Marino, BDV, V Truong, JW Munger, and R Gyimah. 2020. "Direct Measurement Forest Carbon Protocol: A Commercial System-of-Systems to Incentivize Forest Restoration and Management." PEERJ 8 (April). https://doi.org/10.7717/peerj.8891.

Marks, AB. 2020. "(Carbon) Farming Our Way Out Of Climate Change." DENVER LAW REVIEW 97 (3): 497–556.

Martes, L, and M Kohl. 2022. "Improving the Contribution of Forests to Carbon Neutrality under Different Policies-A Case Study from the Hamburg Metropolitan Area." SUSTAINABILITY 14 (4). https://doi.org/10.3390/su14042088.

McAfee, K. 2022. "Shall the American Association of Geographers Endorse Carbon Offsets? Absolutely Not!" PROFESSIONAL GEOGRAPHER 74 (1): 171–77. https://doi.org/10.1080/00330124.2021.1934879.

McArthur, JJ. 2020. "Rethinking Ventilation: A Benefit-Cost Analysis of Carbon-Offset Increased Outdoor Air Provision." BUILDING AND ENVIRONMENT 169 (February). https://doi.org/10.1016/j.buildenv.2019.106551.

Opher, T, M Duhamel, D Posen, DK Panesar, R Brugmann, A Roy, R Zizzo, L Sequeira, A Anvari, and HL MacLean. 2021. "Life Cycle GHG Assessment of a Building Restoration: Case Study of a Heritage Industrial Building in Toronto, Canada." JOURNAL OF CLEANER PRODUCTION 279 (January). https://doi.org/10.1016/j.jclepro.2020.123819.

Orta-Salazar, C, CA Aguirre-Salado, H Reyes-Hernandez, JA Reyes-Aguero, and CA Munoz-Robles. 2021. "Mapping Potential Areas for Conservation Under Forest Carbon Credit Eligibility in a Natural Protected Area in Northern Mexico." TROPICAL CONSERVATION SCIENCE 14 (June). https://doi.org/10.1177/19400829211029448.

Pradhan, BB, RM Shrestha, A Pandey, and B Limmeechokchai. 2018. "Strategies to Achieve Net Zero Emissions in Nepal." CARBON MANAGEMENT 9 (5): 533–48. https://doi.org/10.1080/17583004.2018.1536168.

Prudhomme, R, C O'Donoghue, M Ryan, and D Styles. 2021. "Defining National Biogenic Methane Targets: Implications for National Food Production & Climate Neutrality Objectives." JOURNAL OF ENVIRONMENTAL MANAGEMENT 295 (October). https://doi.org/10.1016/j.jenvman.2021.113058.

Rawhouser, H, ME Cummings, and SR Hiatt. 2019. "DOES A COMMON MECHANISM ENGENDER COMMON RESULTS? SUSTAINABLE DEVELOPMENT TRADE-OFFS IN THE GLOBAL CARBON OFFSET MARKET." ACADEMY OF MANAGEMENT DISCOVERIES 5 (4): 514–29. https://doi.org/10.5465/amd.2018.0160.

Redowan, M, S Phinn, C Roelfsema, and AA Aziz. 2019. "REDD plus Project Design Study for Quantifying Activity Data for Historic Forest Degradation in a Bangladesh Forest Using Landsat Data." JOURNAL OF APPLIED REMOTE SENSING 13 (4). https://doi.org/10.1117/1.JRS.13.046518.

———. 2021. "Satellite Estimation of Emission Factors and Emissions of Biomass-Carbon Due to Deforestation and Forest Degradation in Bangladesh for a REDD plus Program." JOURNAL OF APPLIED REMOTE SENSING 15 (1). https://doi.org/10.1117/1.JRS.15.014510.

Regan, CM, JD Connor, DM Summers, C Settre, PJ O'Connor, and TR Cavagnaro. 2020. "The Influence of Crediting and Permanence Periods on Australian Forest-Based

Carbon Offset Supply.” LAND USE POLICY 97 (September). https://doi.org/10.1016/j.landusepol.2020.104800.

Ritchie, BW, A Kemperman, and S Dolnicar. 2021. “Which Types of Product Attributes Lead to Aviation Voluntary Carbon Offsetting among Air Passengers?” TOURISM MANAGEMENT 85 (August). https://doi.org/10.1016/j.tourman.2020.104276.

Rogerson, RJ, D Horgan, and JJ Roberts. 2021. “Integrating Artificial Urban Wetlands Into Communities: A Pathway to Carbon Zero?” FRONTIERS IN BUILT ENVIRONMENT 7 (December). https://doi.org/10.3389/fbuil.2021.777383.

Rotaris, L, M Giansoldati, and M Scorrano. 2020. “Are Air Travellers Willing to Pay for Reducing or Offsetting Carbon Emissions? Evidence from Italy.” TRANSPORTATION RESEARCH PART A-POLICY AND PRACTICE 142 (December): 71–84. https://doi.org/10.1016/j.tra.2020.10.014.

Sanjuan, MA, C Argiz, P Mora, and A Zaragoza. 2020. “Carbon Dioxide Uptake in the Roadmap 2050 of the Spanish Cement Industry.” ENERGIES 13 (13). https://doi.org/10.3390/en13133452.

Setyowati, AB. 2020. “Making Territory and Negotiating Citizenship in a Climate Mitigation Initiative in Indonesia.” DEVELOPMENT AND CHANGE 51 (1): 144–66. https://doi.org/10.1111/dech.12541.

Sharma, A, SK Jakhar, and TM Choi. 2021. “Would CORSIA Implementation Bring Carbon Neutral Growth in Aviation? A Case of US Full Service Carriers.” TRANSPORTATION RESEARCH PART D-TRANSPORT AND ENVIRONMENT 97 (August). https://doi.org/10.1016/j.trd.2021.102839.

Shirizadeh, B, and P Quirion. 2021. “Low-Carbon Options for the French Power Sector: What Role for Renewables, Nuclear Energy and Carbon Capture and Storage.” ENERGY ECONOMICS 95 (March). https://doi.org/10.1016/j.eneco.2020.105004.

Shubbar, A, M Nasr, M Falah, and Z Al-Khafaji. 2021. “Towards Net Zero Carbon Economy: Improving the Sustainability of Existing Industrial Infrastructures in the UK.” ENERGIES 14 (18). https://doi.org/10.3390/en14185896.

Spanos, I, M Kucukvar, TC Bell, A Elnimah, H Hamdan, B Al Meer, S Prakash, O Lundberg, AA Kutty, and AHA AlKhereibi. 2022. “How FIFA World Cup 2022 (TM) Can Meet the Carbon Neutral Commitments and the United Nations 2030 Agenda for Sustainable Development?: Reflections from the Tree Nursery Project in Qatar.” SUSTAINABLE DEVELOPMENT 30 (1): 203–26. https://doi.org/10.1002/sd.2239.

Streck, C. 2020. “Who Owns REDD plus ? Carbon Markets, Carbon Rights and Entitlements to REDD plus Finance.” FORESTS 11 (9). https://doi.org/10.3390/f11090959.

Stuart, D, R Gunderson, and B Petersen. 2019. “Climate Change and the Polanyian Counter-Movement: Carbon Markets or Degrowth?” NEW POLITICAL ECONOMY 24 (1): 89–102. https://doi.org/10.1080/13563467.2017.1417364.

Udas, E, M Wolk, and M Wilmking. 2018. “The ‘Carbon-Neutral University’ - a Study from Germany.” INTERNATIONAL JOURNAL OF SUSTAINABILITY IN HIGHER EDUCATION 19 (1): 130–45. https://doi.org/10.1108/IJSHE-05-2016-0089.

Uzelgun, MA, M Fernandes-Jesus, and O Kucukural. 2021. “Reception of Climate Activist Messages by Low-Carbon Transition Actors: Argument Evasion in the Carbon Offsetting Debate.” ARGUMENTATION AND ADVOCACY. https://doi.org/10.1080/10511431.2021.1971381.

Wang, ZW, KG Burra, MJ Zhang, XQ Li, XF He, TZ Lei, and AK Gupta. 2020. “Syngas Evolution and Energy Efficiency in CO2-Assisted Gasification of Pine Bark.” APPLIED ENERGY 269 (July). https://doi.org/10.1016/j.apenergy.2020.114996.

Watt, R. 2021. “The Fantasy of Carbon Offsetting.” ENVIRONMENTAL POLITICS 30 (7): 1069–88. https://doi.org/10.1080/09644016.2021.1877063.

Weyrens, JP, O Therasme, and RH Germain. 2022. “Quantifying the Life Cycle Greenhouse Gas Emissions of a Mechanized Shelterwood Harvest Producing Both Sawtimber and Woodchips.” FORESTS 13 (1). https://doi.org/10.3390/f13010070.

Winchester, N. 2019. “A Win-Win Solution to Abate Aviation CO2 Emissions.” JOURNAL OF AIR TRANSPORT MANAGEMENT 80 (September). https://doi.org/10.1016/j.jairtraman.2019.101692.

Wiryadinata, S, J Morejohn, and K Kornbluth. 2019. “Pathways to Carbon Neutral Energy Systems at the University of California, Davis.” RENEWABLE ENERGY 130 (January): 853–66. https://doi.org/10.1016/j.renene.2018.06.100.

Wolf, SA, and R Ghosh. 2020. “A Practice-Centered Analysis of Environmental Accounting Standards: Integrating Agriculture into Carbon Governance.” LAND USE POLICY 96 (July). https://doi.org/10.1016/j.landusepol.2018.08.003.

Woolf, D, J Lehmann, S Ogle, AW Kishimoto-Mo, B McConkey, and J Baldock. 2021. “Greenhouse Gas Inventory Model for Biochar Additions to Soil.” ENVIRONMENTAL SCIENCE & TECHNOLOGY 55 (21): 14795–805. https://doi.org/10.1021/acs.est.1c02425.

Xu, YY, and S Salem. 2021. "Explosive Behaviors in Chinese Carbon Markets: Are There Price Bubbles in Eight Pilots?" RENEWABLE & SUSTAINABLE ENERGY REVIEWS 145 (July). https://doi.org/10.1016/j.rser.2021.111089.

Yao, SY, XY Yu, S Yan, and SY Wen. 2021. "Heterogeneous Emission Trading Schemes and Green Innovation." ENERGY POLICY 155 (August). https://doi.org/10.1016/j.enpol.2021.112367.

Zhang, JR, SJ Zhang, RX Wu, MS Duan, D Zhang, Y Wu, and JM Hao. 2021. "The New CORSIA Baseline Has Limited Motivation to Promote the Green Recovery of Global Aviation." ENVIRONMENTAL POLLUTION 289 (November). https://doi.org/10.1016/j.envpol.2021.117833.

Zhang, XY, J Gu, and Y Liu. 2022. "Necessity of Direct Energy and Ammonium Recovery for Carbon Neutral Municipal Wastewater Reclamation in an Innovative Anaerobic MBR-Biochar Adsorption-Reverse Osmosis Process." WATER RESEARCH 211 (March). https://doi.org/10.1016/j.watres.2022.118058.

Zhao, LT, J Miao, S Qu, and XH Chen. 2021. "A Multi-Factor Integrated Model for Carbon Price Forecasting: Market Interaction Promoting Carbon Emission Reduction." SCIENCE OF THE TOTAL ENVIRONMENT 796 (November). https://doi.org/10.1016/j.scitotenv.2021.149110.
